# mTORC1 activation induces B7-H3-dependent metabolic reprogramming in renal cell carcinoma (RCC)

**DOI:** 10.1038/s41388-026-03891-w

**Published:** 2026-07-20

**Authors:** Keying Li, Heng Du, Yifan Wang, Damir Khabibullin, Michel Alchoueiry, Melissa Daou, John M. Asara, David J. Kwiatkowski, Elizabeth P. Henske, Heng-Jia Liu

**Affiliations:** 1 Department of Colorectal Surgery of Sir Run Run Shaw Hospital, and Centre for Infection, Immunity and Cancer of Zhejiang University-University of Edinburgh Institute (ZJE), Zhejiang University School of Medicine, Zhejiang University, Hangzhou, PR China; 2 Department of Thoracic Surgery, West China Hospital, Sichuan University, Chengdu, PR China; 3 Lung Cancer Center, West China Hospital, Sichuan University, Chengdu, PR China; 4 Pulmonary and Critical Care Medicine, Department of Medicine, Brigham and Women’s Hospital and Harvard Medical School, Boston, MA USA; 5 Division of Signal Transduction, Beth Israel Deaconess Medical Center, Boston, MA USA; 6 Department of Medicine, Harvard Medical School, Boston, MA USA; 7 ZJE Cross Lung Research Alliance, International Joint Center for Biomedical Sciences (Haining), Zhejiang University, Haining, PR China; 8 Edinburgh Medical School, College of Medicine and Veterinary Medicine, The University of Edinburgh, Edinburgh, UK

**Keywords:** Cancer metabolism, Cancer models

## Abstract

Renal cell carcinoma (RCC) affects over 435,000 people worldwide each year and remains a significant cause of cancer mortality. Aberrant activation of pathways such as mTORC1 is frequently observed in RCC, yet the mechanisms by which mTORC1 contributes to tumor development remain poorly understood. The lack of suitable animal models has limited progress in uncovering these mechanisms and advancing targeted treatments. Here, we performed single-cell transcriptomic, proteomic, and metabolomic profiling of kidneys from mice with embryonic *Tsc2* deletion at E17.5, which revealed a distinct tumor cell population characterized by intercalated cell signatures, co-expression of *Foxi1* and *Rhcg*, and hyperactivated mTORC1. These lesions closely resemble human chromophobe renal cell carcinoma (ChRCC), a subtype of RCC. The tumors show marked upregulation of the immune checkpoint molecule B7-H3. Mechanistically, the transcription factor NRF1 is elevated in *Tsc2*-deficient lesions and drives B7-H3 expression in tumor cells. In human specimens, B7-H3 is broadly expressed in both classical and sarcomatoid ChRCC and correlates with hallmark markers FOXI1, MUC2, and HEPACAM2. Strikingly, B7-H3 deletion in mice suppresses *Tsc2* loss-driven cystic and tumor formation, restores amino acid metabolism, and attenuates aminoacyl-tRNA biosynthesis and proteasome activity. Collectively, these findings identify B7-H3 as a functional driver of *Tsc2*-mediated ChRCC and a promising therapeutic target in TSC-associated renal tumors.

## Introduction

Renal cell carcinoma (RCC) represents a heterogeneous group of malignancies that arise from the renal parenchyma and encompasses more than 20 histologically and molecularly distinct subtypes of epithelial origin [1]. Epithelial-derived RCCs are further classified into seven major subtypes: clear cell RCC (ccRCC), papillary RCC (pRCC), chromophobe RCC (ChRCC), oncocytoma, collecting duct carcinoma (CDC), renal medullary carcinoma, and translocation-associated RCCs [1]. ChRCC is the third most common subtype and exhibits unique features [2], though its pathogenesis remains poorly understood. Genomic studies have implicated mTORC1 hyperactivation, often due to PTEN loss and mitochondrial genome dysregulation, in ChRCC development [3–5]. Therapeutic vulnerabilities, such as targeting the glutathione salvage pathway and ferroptosis, have been proposed [6, 7], but findings are limited to in vitro and subcutaneous xenograft models that do not replicate the native kidney environment, highlighting the lack of reliable preclinical models.

ChRCC is also associated with genetic syndromes, including tuberous sclerosis complex (TSC) and Birt-Hogg-Dubé (BHD) syndrome [8, 9]. In TSC, mutations in *TSC1* or *TSC2* genes disrupt mTORC1 signaling and contribute to the development of ChRCC and other epithelial renal tumors, which occur in ~5% of TSC patients [10]. Among all epithelial renal tumor subtypes in TSC patients, 33–59% are classified as chromophobe-like RCCs or hybrid oncocytic chromophobe tumors (HOCT) [11, 12]. Similarly, BHD, caused by mutations in the *FLCN* gene, predisposes individuals to ChRCC and HOCT. While kidney-specific or heterozygous Flcn knockout (KO) mice develop ChRCC-like or HOCT [13–16], ChRCC has not been observed in previously reported TSC KO mouse models, which typically develop renal cysts and cystadenomas [17–23].

Immune checkpoint molecules, critical regulators of immune responses, are essential for maintaining self-tolerance and preventing autoimmunity, and are frequently exploited by tumors to evade immune surveillance [24]. Among these, the B7 family of ligands plays a central role in modulating T cell activity through both co-stimulatory and co-inhibitory interactions with CD28 family receptors [25, 26]. *CD276*, also known as B7-H3, is a type I transmembrane protein and member of the B7 family [27, 28]. Although initially identified as a co-stimulatory molecule, B7-H3 is now predominantly recognized for its immunosuppressive function and contribution to tumor immune evasion [29–31]. B7-H3 is overexpressed across a broad spectrum of malignancies, and its expression is often associated with poor clinical outcomes, positioning it as an attractive target for cancer immunotherapy [32–39]. In the tumor microenvironment, B7-H3 impairs adaptive immune responses by inhibiting the activity of cytotoxic CD8⁺ T cells, CD4⁺ T cells and natural killer (NK) cells, thereby promoting an immunosuppressive milieu [40–43]. Our prior studies discovered TSC2 loss upregulated B7-H3 expression through mTORC1/S6K/YY2 signaling, thereby inhibiting the activity of cytotoxic CD38^+^CD39^+^CD4^+^ T cells [31]. Beyond its immunological functions, B7-H3 also promotes tumor progression through non-immune mechanisms, including activation of NF-κB and mTOR signaling pathways to enhance angiogenesis [44, 45]. Additionally, B7-H3 has been implicated in regulating lipid metabolism via the SREBP1/FASN axis and stabilizing HIF-1α, leading to increased reactive oxygen species (ROS) and glycolytic flux [46, 47]. The role of B7-H3 in modulating tumor metabolic and proteasomal networks remains largely unexplored.

We generated a conditional mouse model by disrupting the *Tsc2* gene at embryonic day 17.5. These mice develop a spectrum of kidney lesions, including cysts, papillary cystadenomas, and chromophobe-like RCC. Single-cell transcriptomic profiling identified a distinct cyst/tumor cell population with intercalated cell signatures and elevated mTORC1 activity. Further analysis revealed transcriptional programs and pathways characteristic of human ChRCC, including expression of FOXI1, HEPACAM2, and MUC20. B7-H3 is expressed in both classical and sarcomatoid human ChRCC specimens and correlates with the hallmark markers FOXI1, MUC20, and HEPACAM2. In the kidneys of *Tsc2*-inactivated mice, NRF1 is upregulated, thereby promoting B7-H3 transcription and resulting in its marked overexpression in cysts and tumors. Genetic ablation of B7-H3 significantly reduced *Tsc2* loss-associated cystic and neoplastic growth. Integrated multi-omic analysis further showed that *Tsc2* inactivation enhances amino acid metabolism and aminoacyl-tRNA biosynthesis, both of which are normalized by B7-H3 deletion, thereby establishing B7-H3 as a critical effector in *Tsc2*-associated renal tumorigenesis.

## Methods

### Study approval

In vivo genetic models: All mouse work were approved by the Zhejiang University-University of Edinburgh Institute Laboratory Animal Centre (approval: ZJU20240699) and the Brigham and Women’s Hospital Institutional Animal Care and Use Committee (approval: 2019N000158). Immunohistochemistry and hematoxylin-eosin staining: All studies involving the use of human specimens were approved by the Human Ethics Committee of West China Hospital, Sichuan University (approval: 2026-666). Written informed consent was obtained from all patients prior to participation in the study. This manuscript does not contain any identifiable images of human participants; therefore, no additional consent for publication of images was required. All experiments were performed in accordance with the relevant guidelines and regulations.

### Mice

Rosa26-CreERT2 mice were purchased from Jackson Laboratory. *Tsc2*^*flox/flox*^ mice were a gift from Dr. Michael Gambello. B7-H3-deficient (*Cd276*^*−/−*^) mice in pure C57BL/6 background were a gift from Dr. Tak W. Mak. The generation and genotyping of B7-H3 knockout mice are described elsewhere [41]. Rosa26-CreERT2 mice were first crossed with *Tsc2*^*flox/flox*^ mice, then with *Cd276*^*−/−*^ mice to generate Rosa26-CreERT2 *Tsc2*^*flox/flox*^ *Cd276*^*+/+*^ and Rosa26-CreERT2 *Tsc2*^*flox/flox*^ *Cd276*^*−/−*^ mice. All the mice used in these experiments were at least 88% C57BL/6 J background, with control mice being vehicle (corn oil)-treated littermates of mutant mice. Both male and female mice were used. Knockout efficiency was determined by PCR and agarose gel electrophoresis, and the primers are shown in Table S1.

### Animal studies

Tamoxifen (10 mg/ml, Sigma-Aldrich, Cat#T5648) dissolved in corn oil was injected i.p. into pregnant females with a dose of 10 mg/kg at E17.5 (the day of vaginal plug observed as considered as E0.5). All mice were housed in an animal facility with a 12 h light/12 h dark cycle at 72 °F and 40% humidity with unrestricted access to standard chow and water, except for mice undergoing blood chemistry analysis, which were starved for 6 h before the collection of tissues. For all the animal studies, we have chosen to use at least three mice per group to ensure adequate power to detect difference between groups.

## Results

### Conditional embryonic TSC2 knockout leads to multisystem abnormalities, including kidney cysts and solid tumors

**Fig. 1:**
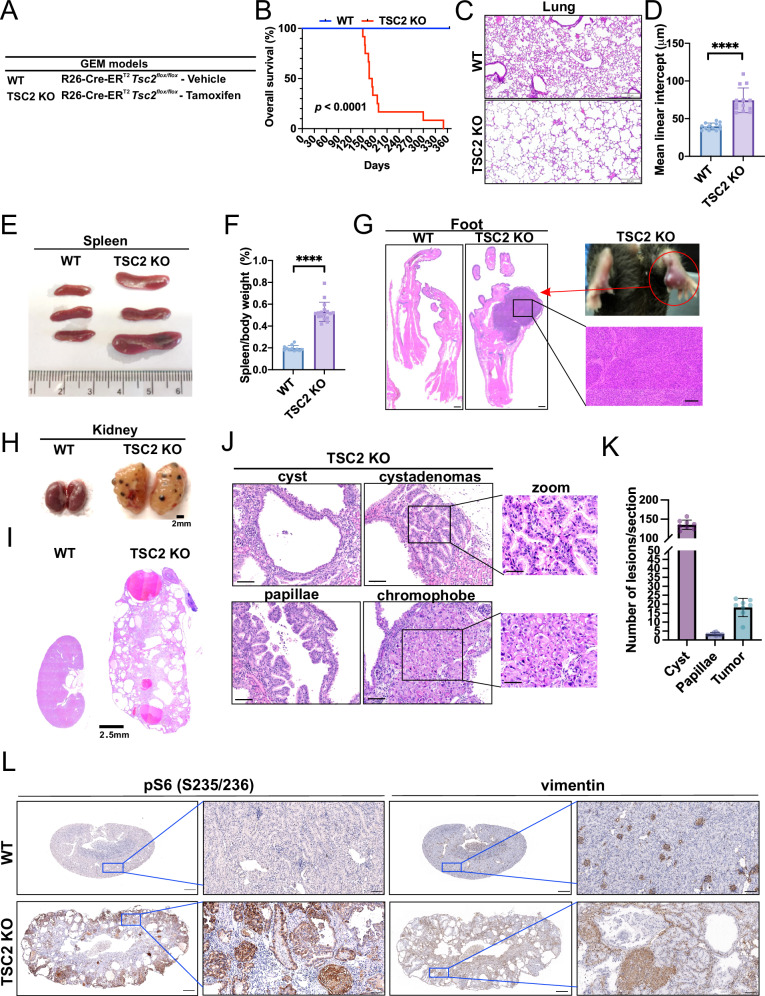
*Tsc2* loss triggers mTORC1-driven multisystem abnormalities, renal cysts, and solid tumors. **A** Table summarizing the transgenic mouse models and their genotypes. Tamoxifen (10 mg per kg body weight) or vehicle was i.p. administered to pregnant females at E17.5. **B** Kaplan–Meier plot of survival in WT (*n* = 17) and TSC2 KO (*n* = 12) mice. **C** Representative H&E images of lung sections from 24-week-old mice of the indicated genotypes. Scale bar = 200 μm. **D** Quantification of mean linear intercept of alveolar spaces in the lungs from WT (*n* = 13) and TSC2 KO (*n* = 13) mice. Data are presented as mean ± SD, analyzed using two-tailed Mann–Whitney test; ****p < 0.0001. **E** Representative spleens from 24-week-old WT and TSC2 KO mice. **F** Quantification of the spleen weight-to-body weight ratio in WT (*n* = 11) and TSC2 KO (*n* = 17) mice. Data are presented as mean ± SD, analyzed using two-tailed Mann–Whitney test; ****p < 0.0001. **G** Representative H&E images of the foot from WT and TSC2 KO mice (left), a representative photograph of the feet from a TSC2 KO mouse (top right), and a higher-magnification H&E image of angiomyosarcoma in the foot of a TSC2 KO mouse (bottom right). Scale bars = 1 mm (left images); 200 μm (bottom right image). **H** Representative kidneys from 24-week-old WT and TSC2 KO mice. Scale bar = 2 mm. **I** Representative H&E images of whole kidney sections from 24-week-old mice of the indicated genotypes. Scale bar = 2.5 mm. **J** Representative H&E images of kidney pathological features from TSC2 KO mice. Scale bar = 200 μm. **K** Quantification of kidney lesion numbers from TSC2 KO mice by counting 10 sections/kidney (*n* = 8). Mean ± SD. **L** Representative images of pS6 (S235/236) and vimentin immunohistochemical staining on kidney sections from TSC2 KO mice (*n* = 3/group). Scale bar = 100 μm.

We generated mice that are homozygous for a floxed *Tsc2* allele (*Tsc2*^*flox/flox*^) and carry a ubiquitously expressed and temporally promoter-regulated Cre transgenic allele (GtROSA26-CreER^T2^ [48]). To mitigate the embryonic lethality resulting from complete knockout of the *Tsc2* gene throughout the body as previously described [49], we administered a low dose of tamoxifen intraperitoneally (i.p., 10 mg/kg) to pregnant female mice on embryonic day 17.5 (E17.5) (referred to as TSC2 KO; Fig. 1A). TSC2 KO offspring mature into adults at the expected Mendelian ratio compared to control littermates. However, >50% of TSC2 KO mice died by age 165 days (Fig. 1B). Consequently, our analysis focused on 5–6-month-old mice before symptoms appeared. We quantified recombination efficiency following tamoxifen administration across multiple tissues. The recombination efficiency is approximately 42% in kidney tissue (the primary organ of interest) and ranges from 12–72% in other examined tissues, with minimal or undetectable recombination in the brain and bone (Supplementary Fig. 1A, B). Consistent with the lack of recombination in the brain, the mice do not develop seizures. Based on histopathological and functional analyses, the observed lethality is attributable to severe kidney manifestations rather than extra-renal involvement. We examined major organs in TSC2 KO mice and found that these mice exhibited enlargement of alveolar spaces in the lungs, with the mean linear intercept 1.9-fold higher in TSC2 KO mice than in WT controls (*P* < 0.0001; Fig. 1C, D). TSC2 KO mice also showed splenomegaly, with spleen size increased by 2.7-fold relative to WT mice (*P* < 0.0001, Fig. 1E, F). In addition, all late-stage mice (approximately 5–6 months old) developed paw angiosarcoma (Fig. 1G). Most importantly, TSC2 KO mice have enlarged cystic kidneys, with hemorrhage present in some cysts (Fig. 1H, I). The observation of cystic kidney enlargement and hemorrhage was reported in a previous study, in which *Tsc1* was knocked out at E15.5 using a ubiquitous Cre transgenic allele (*CAGGCre-ER*^*TM*^) [19]. However, those *Tsc1* mice did not survive beyond 10 weeks of age. This difference may be due to the timing of tamoxifen induction, the Cre driver used, and/or the distinct roles of *Tsc1* and *Tsc2* [50, 51]. The cysts within the TSC2 KO kidneys are heterogeneous; some have a single layer of epithelium, while others have papillary projections; both phenotypes are similar to those previously observed in *Tsc2*^+/−^ and *Tsc1*^*+/−*^ mice. In addition, two types of solid tumors are seen: one resembling the cystadenomas observed in the kidneys of *Tsc2*^*+/−*^ and *Tsc1*^*+/−*^ mice [17, 19, 23], and the other resembling chromophobe RCC, with large polygonal cells characterized by abundant, pale cytoplasm and irregular nuclei with perinuclear halos seen in some cells (Fig. 1J). On average, each kidney section contained 122 cysts, 2 papillae, and 18 solid tumors (Fig. 1K). Immunostaining with phospho-S6^S235,236^ antibody, a marker of mTORC1 hyperactivity, revealed that cysts, papillae, and tumors display high mTORC1 signaling. Interestingly, staining with the mesenchymal marker vimentin showed that only the solid tumors, and not the cystadenoma, were positive (Fig. 1L).

### *Tsc2* loss drives cyst/tumor formation from a type A intercalated cell lineage

**Fig. 2:**
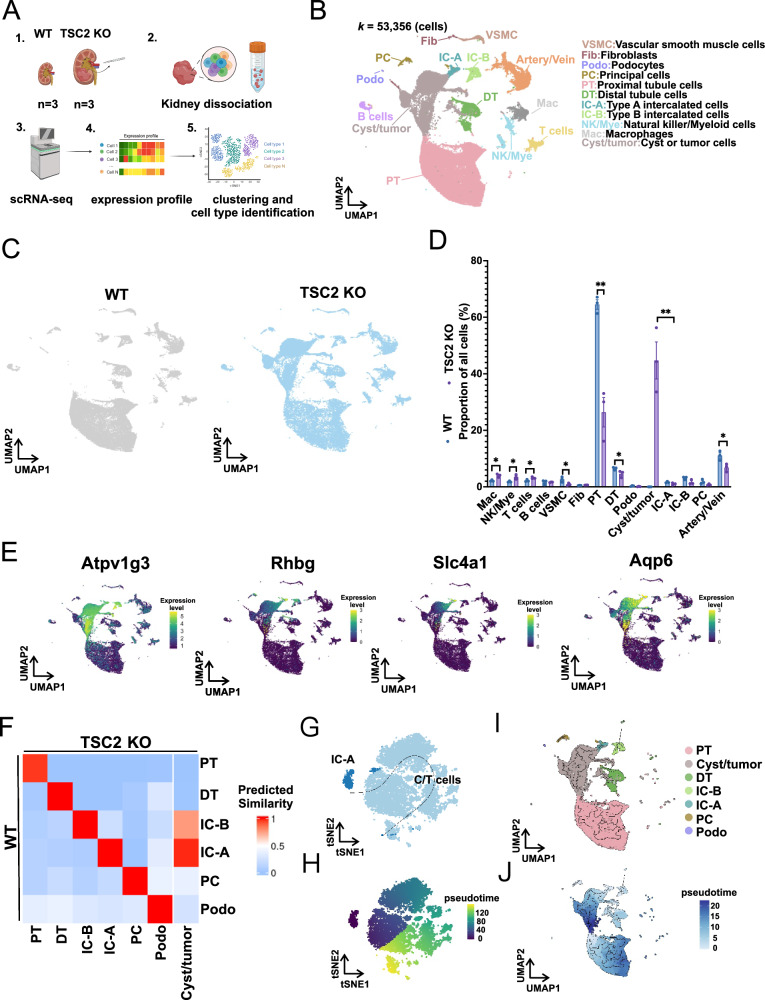
*Tsc2* loss drives expansion of C/T cells originating from type A intercalated cells. **A** Schematic of the study design. Kidneys from 24-week-old WT or TSC2 KO mice were processed for scRNA-seq, followed by data processing and analysis (*n* = 3/group). **B** UMAP plot of a total of 53,356 cells from 24-week-old WT and TSC2 KO kidneys. Cells were assigned to fourteen cell types based on their marker expression. PC principal cells, Podo podocytes, PT proximal Tubule, IC-A type A Intercalated cells, IC-B type B Intercalated cells, NK/Mye NK/myeloid cells, DT distal tubule, Fib fibroblasts, Mac macrophages, VSMC vascular smooth muscle cells. **C** UMAP atlases split by indicated genotypes. **D** Bar plot showing the comparison of the distribution of different cell clusters between WT and TSC2 KO kidneys. (*n* = 3/group), mean ± SD, two-way ANOVA with Tukey’s multiple comparisons test, *p < 0.05, **p < 0.001. **E** Feature plots of expression of intercalated cell marker genes *Atpv1g3*, *Aqp6*, *Rhbg*, and *Slc4a1* in the kidneys of 24-week-old WT and TSC2 KO mice. **F** Heatmap showing predicted similarities among epithelial cell subtypes from combined TSC2 KO and WT samples, calculated by a machine learning model trained on scRNA data from epithelial cells of WT kidneys. tSNE plot showing the pseudotime trajectory of cyst/tumor cells and type A intercalated cells split by clusters (top, **G**) and pseudotimes (bottom, **H**). The curve represents the developmental ordering of the two cell types as defined by Slingshot. UMAP plots showing the pseudotime trajectory of the indicated cell types, colored by clusters (**I**) and pseudotime (**J**). The curve represents the inferred developmental ordering of the cell types, as defined by Monocle 3.

To investigate the impact of TSC2 loss on kidney cellular composition, we performed single-cell RNA sequencing (scRNA-seq) on kidneys from 24-week-old wild-type (WT) and TSC2 knockout (KO) mice (*n* = 3 per group) (Fig. 2A). Following sequencing, stringent quality control measures were applied to eliminate doublets and low-quality cells, ensuring a robust correlation between gene count and UMI number (Supplementary Fig. 2A). In total, we obtained 53,356 high-quality single-cell transcriptomes, 25,106 from WT mice and 28,250 from TSC2 KO mice (Fig. 2B and Supplementary Fig. 2B). Batch effects were mitigated using reciprocal PCA (RPCA)-based data integration [52]. Unsupervised clustering combined with differential gene expression analysis enabled the identification of 14 distinct kidney cell types. These included proximal tubule (PT), distal tubules (DT), type A intercalated cells (IC-A), type B intercalated cells (IC-B), principal cells (PC), podocytes (Podo), fibroblasts (Fib), vascular smooth muscle cells (VSMCs), arterial/venous endothelial cells (Artery/Vein), macrophages (Mac), T cells, NK/myeloid cells (NK/Mye), B cells, and cyst/tumor cells. Cell types are assigned based on the enrichment of gene sets corresponding to established kidney cell-type-specific markers [53] (Fig. 2B and Supplementary Fig. 2C). Clusters that specifically express *Slc4a1* are annotated as IC-A, while clusters expressing Slc26a4 are defined as IC-B (Supplementary Fig. 2C).

The cyst/tumor cell population is detected exclusively in TSC2 KO kidneys and is absent in WT samples (Fig. 2C). As expected, PT represented the majority (64%) of sequenced cells in WT kidneys. In contrast, their proportion decreased to 27% in TSC2 KO kidneys, largely due to the emergence of cyst/tumor cells, which accounted for 45% of all cells in these samples (Fig. 2D). In addition, we performed co-staining of Cre with the proximal tubule marker LRP2. Cre expression is detected broadly across all cell types, including LRP2-positive proximal tubule (PT) cells, whereas LRP2 is not expressed in cyst/tumor cells (Supplementary Fig. 3A). These findings indicate that the expansion of the cyst/tumor population is unlikely to result from preferential Cre activity in proximal tubule cells. To further confirm Cre-mediated recombination, we performed co-staining for TSC2 and LRP2. In cyst/tumor-adjacent normal kidney regions of TSC2 KO mice, LRP2-positive cells retained TSC2 expression. In contrast, cells within cyst/tumor regions lacked TSC2 expression, consistent with effective Cre-mediated deletion of the *Tsc2* gene. These results suggest that cyst/tumor cells originate from cells that have undergone *Tsc2* deletion (Supplementary Fig. 3B). Moreover, co-staining of Ki-67 and phospho-S6^S235,236^ revealed co-expression in approximately 6.3% of the cyst/tumor cells, indicating that increased proliferation occurs specifically in mTOR-positive cells (Supplementary Fig. 3C), supporting that both selective mTOR activation and enhanced proliferation contribute to the observed expansion of the cyst/tumor cell population. Additionally, the proportions of macrophages, NK/myeloid, and T cells are elevated in TSC2 KO kidneys (Fig. 2D). The cyst/tumor cells exhibited high expression of intercalated cell markers, including *Atp6v1g3*, *Aqp6*, *Rhcg*, and *Slc4a1* (Fig. 2E), suggesting a possible origin from intercalated cells. To determine the cellular origin of cyst/tumor cells, we first applied a previously developed machine learning approach, glmnet [54], to identify the cell of origin. Using scRNA-seq data of epithelial cells from WT kidneys in our cohort as the training set, we developed a model to predict the identities of distinct kidney epithelial cell types based on their transcriptomic profiles. The model achieved high accuracy in classifying cell types, with strong predicted similarity scores (>0.8, range 0–1) across all types. We then tested the model on scRNA-seq data from TSC2 KO kidneys, including all epithelial cell types and cyst/tumor cells. The cyst/tumor cells showed the highest predicted similarity to type A intercalated cells (IC-A) (Fig. 2F). Furthermore, we reconstructed the developmental trajectory of IC-A and cyst/tumor cells using Slingshot [55] for pseudotime analysis. The results indicated that IC-A progressed toward cyst/tumor cells along the pseudotime scale (Fig. 2G, H). We performed additional pseudotime analyses, including all kidney epithelial and cyst/tumor cells. When kidney epithelial cells were included, the overall trajectory structure remained largely consistent, the cyst/tumor cell population still aligned closely with type A intercalated cells (IC-A) (Fig. 2I, J), supporting our original inference. Notably, pseudotime analysis starting from PC produced trajectories that did not converge onto the cyst/tumor cell population, consistent with the observation that L1cam-positive cells follow distinct differentiation paths compared with Foxi1-positive cells (Fig. 2I, J). These analyses reinforce that the cyst/tumor cell population likely derives from IC-A rather than principal cells (PC), supporting the model that cysts, cystadenomas, and chromophobe carcinomas originate from a common IC-A lineage. Interestingly, we found that distal tubule (DT) cells may also contribute to the cyst/tumor cell population, as they also project onto the cyst/tumor cell population (Fig. 2I, J).

Gene expression analysis was performed by comparing each of the 14 cell types between TSC2 KO and WT kidney cells. Macrophages exhibited the highest number of differentially expressed genes (DEGs), with 293 in total (211 upregulated, 82 downregulated), followed by NK/myeloid cells, which displayed 245 DEGs (40 upregulated, 205 downregulated) (Supplementary Fig. 2D). The expression levels of *Atp6v1g3*, *Aqp6*, *Rhcg*, *Slc4a1*, or *Slc26a4* in the IC-A and IC-B are similar in TSC2 KO compared with the corresponding cell types in WT mice (Supplementary Fig. 2E).

### Kidney tumors develop in TSC2 mice are similar to human ChRCC

**Fig. 3:**
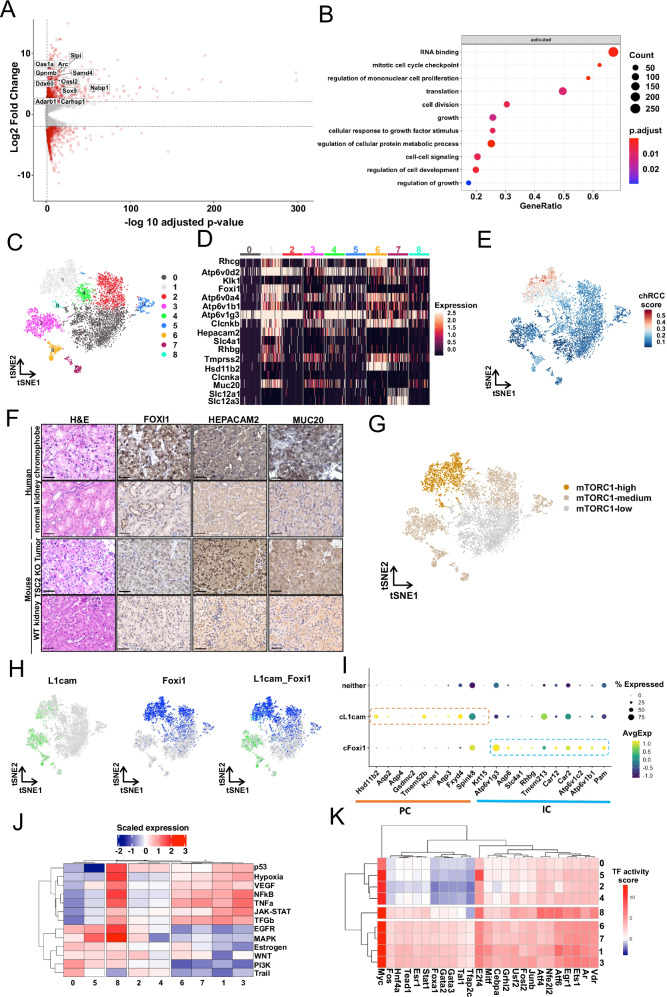
TSC2 KO mice develop renal tumors showing similarities to human ChRCC. **A** Volcano plot showing differentially expressed genes (DEGs) between the cyst/tumor cells from TSC2 KO mice and type A intercalated cells from WT mice. Each dot represents a gene. Genes with absolute log_2_ fold-change of 2 and adjusted *p* value *p* ≤ 0.05 are represented in red. **B** GSEA of cyst/tumor cells from TSC2 KO mice versus type A intercalated cells from WT mice. Enriched gene sets (*p* < 0.05; *p* adjusted value < 0.010) in cyst/tumor cells of TSC2 KO mice were ranked by normalized enrichment score. **C** tSNE plot of cyst/tumor cells showing nine distinct clusters. **D** Heatmap of scaled, normalized counts averaged within cyst/tumor cells for genes differentially expressed between different cyst/tumor cell clusters in TSC2 KO mice. **E** tSNE plot showing ChRCC score calculated by UCell. **F** Representative images of H&E staining and IHC staining for FOXI1, HEPACAM2, and MUC20 in renal tumor sections from ChRCC patients (*n* = 30), tumor-adjacent kidney tissues from ChRCC patients (*n* = 3), and kidneys from WT (*n* = 3) and TSC2 KO (*n* = 3) mice. Scale bar = 50 μm. **G** ChRCC population (cluster 1) shows a high mTORC1 score. **H** tSNE plots showing co-expression of *L1cam* and *Foxi1* in cyst/tumor cells. **I** Dot plots of expression of marker genes for intercalated cell (IC) and principal cell (PC) across clusters of L1cam^+^ (cL1cam), Foxi1^+^ (cFoxi1), and L1cam^−^/Foxi1^−^ cells (neither). **J** Heatmap showing the activity score of the 13 PROGENy pathways in all clusters of cyst/tumor cells. **K** Heatmap displaying the differential DoRothEA transcription factor activity score in the nine clusters of cyst/tumor cells.

Differentially expressed gene and gene set enrichment (GSEA) analyses of the cyst/tumor cells from TSC2 KO mice versus type A intercalated cells (IC-A) from WT mice revealed upregulated genes involved in RNA binding, including *Nabp1*, *Oas1a*, *Slpi*, *Arc*, etc. (Fig. 3A, B). We also observed increased *Gpnmb* expression, which is consistently associated with TSC loss in mouse models, human TSC-associated tumors [56], and human ChRCC [57].

Sub-clustering of the cyst/tumor cell population revealed nine distinct clusters (Fig. 3C). Notably, Cluster 1 exhibited high expression of ChRCC markers, including *Foxi1*, *Hepacam2*, and *Muc20* (Fig. 3D). They are enriched for the ChRCC gene signature previously defined by Lindgren and Nassar et al. [58, 59] (Fig. 3E). Interestingly, Cluster 6 expressed markers characteristic of both intercalated cells (*Rhcg*) and principal cells (*Hsd11b2*) (Fig. 3D), suggesting that these cells may originate from a transitional cell type. Since our pseudotime analysis revealed that distal tubule (DT) cells converge onto cyst/tumor cells, we found that cells in cluster 7 express DT marker genes *Slc12a1* and *Slc12a3* (Fig. 3D), suggesting that a small subset of cyst/tumor cells (cluster 7) may originate from DT cells. Additionally, we performed immunostaining on TSC2 KO mouse kidney tumors and human ChRCC samples using antibodies against FOXI1, HEPACAM2, and MUC20. Consistent with our scRNA-seq data, these markers were robustly expressed in kidney tumors of TSC2 KO mice, supporting a molecular resemblance to human ChRCC (Fig. 3F).

We stratified the nine cyst/tumor cell clusters based on mTORC1 activity scores, revealing that clusters 1 and 8 exhibited the most pronounced gene signature profiles associated with mTORC1 activation (Fig. 3G and Supplementary Fig. 4A). We further identified 19 candidate genes potentially regulated by elevated mTORC1 activity, including *Adgrf5*, *Mme*, *Clcn5*, *Pam*, *Aplp2*, *Itpr2*, *Pcolce*, *Hspa5*, *Hexb*, *Col18a1*, *Dkk2*, *Aqp6*, *Alcam*, *Clcnkb*, *Psap*, *Car12*, *Muc20*, *Kit*, and *Hsp90b1* (Supplementary Fig. 4B, C).

BHD-associated hybrid oncocytic-chromophobe tumors (HOCT) contain two transcriptionally distinct tumor cell populations: L1 cell adhesion molecule (L1CAM)-expressing cells and FOXI1-expressing cells, whose expression patterns are mutually exclusive at the single-cell level [60, 61]. Our single-cell transcriptomic analysis of cyst/tumor cell population revealed discrete clusters of *Foxi1*- and *L1cam*-expressing cells, suggesting a conserved molecular axis between BHD- and TSC-associated renal tumorigenesis (Fig. 3H). Notably, *L1cam*-positive cells express marker genes indicative of principal cells within the collecting duct, whereas *Foxi1*-positive cells express markers representative of intercalated cells (Fig. 3I). These distinct expression programs suggest that such cellular divergence may arise, at least in part, via developmental mechanisms analogous to those governing normal collecting duct differentiation.

PROGENy pathway activity analysis further demonstrated that cells in cluster 1 exhibit robust TGFβ signaling, similar to the pathway activity observed in clusters 3, 6, and 7. By contrast, clusters 0 and 5 were preferentially enriched for EGFR pathway activity. Intriguingly, cluster 8, which harbors the highest mTORC1 activity score, concurrently displayed heightened activation of the JAK-STAT, TGFβ, and EGFR pathways (Fig. 3J and Supplementary Fig. 4D). Transcription factor (TF) regulon analysis revealed that clusters 1, 3, 6, and 7 share highly concordant TF activity signatures (Fig. 3K), further reinforcing the pathway-level insights derived from PROGENy analysis. To further delineate intracluster heterogeneity, we stratified cyst/tumor cell cluster 1 based on the co-expression of *Foxi1* and *Rhcg*. Notably, the Foxi1/Rhcg-positive subpopulation exhibits differential expression of over 1500 genes when compared to other clusters, highlighting a distinct transcriptional program (Supplementary Fig. 4E).

### B7-H3 is highly expressed in human ChRCC, and loss of B7-H3 rescues cyst and tumor formation in TSC2 KO mice

**Fig. 4:**
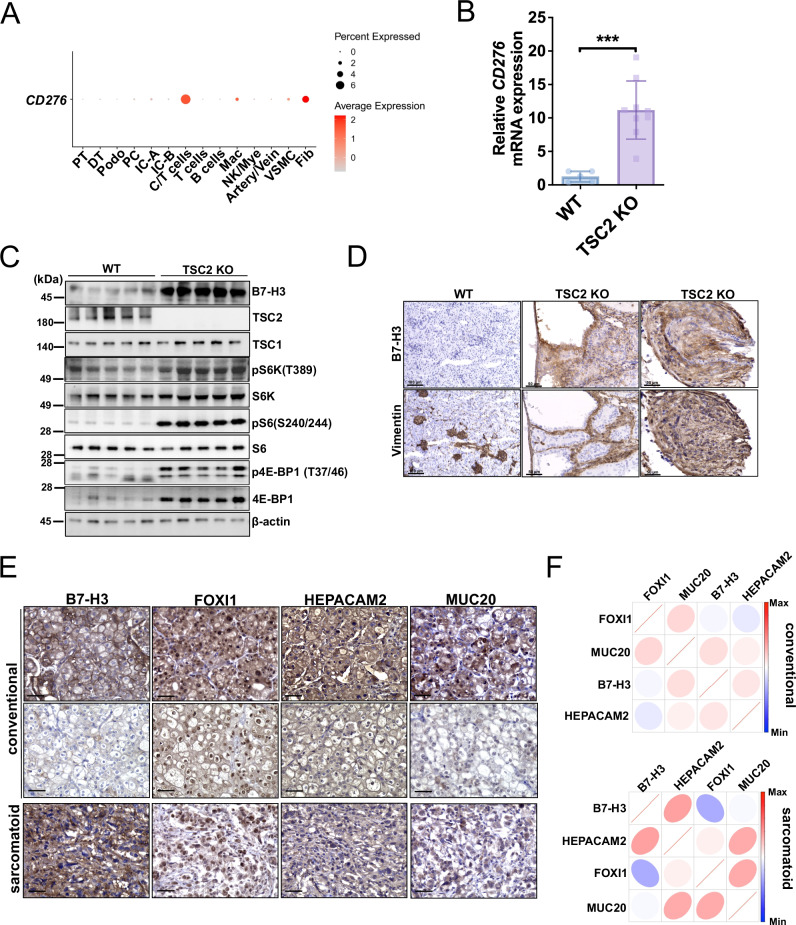
B7-H3 is highly expressed in human ChRCC. **A** Dot plots showing *CD276* expression in combined kidney scRNA-seq data from three 24-week-old WT mice and three TSC2 KO mice. **B** qRT-PCR analysis of *CD276* mRNA expression in mouse kidneys from WT (*n* = 5) and TSC2 KO (*n* = 9) mice. Results are presented as mean ± SD, analyzed using one-way ANOVA test; ***p < 0.001. **C** Immunoblot analysis of whole kidney tissue lysates from WT and TSC2 KO mice (*n* = 5/group). **D** Representative images showing B7-H3 and vimentin immunohistochemistry (IHC) staining from WT and TSC2 KO mice (*n* = 5/group). Left image scale bar = 100 µm; middle and right image scale bar = 50 µm. **E** Representative images of B7-H3, FOXI1, HEPACAM2, and MUC20 IHC staining in 32 conventional and 11 human sarcomatoid ChRCC specimens. scale bar = 50 µm. **F** Correlation analysis of the staining intensity of B7-H3, FOXI1, HEPACAM2, and MUC20 presented in (**E**).

We previously demonstrated that B7-H3/*CD276* expression is strongly upregulated in multiple human and mouse *Tsc2*-deficient cells [31]. The scRNA-seq data from kidney cells of TSC2 KO mice also revealed that cyst/tumor cells exhibit the highest *CD276* expression, followed by fibroblasts (Fib), macrophages (Mac), and vascular smooth muscle cells (VSMCs), while other cell types show minimal *CD276* expression (Fig. 4A). Additionally, immunoblotting and qRT-PCR analyses revealed a marked increase in B7-H3 protein and mRNA levels in the kidneys of TSC2 KO mice (Fig. 4B, C) Consistently, immunohistochemical staining confirmed elevated B7-H3 expression in cystic and tumor cells within the TSC2 KO kidneys (Fig. 4D).

**Fig. 5:**
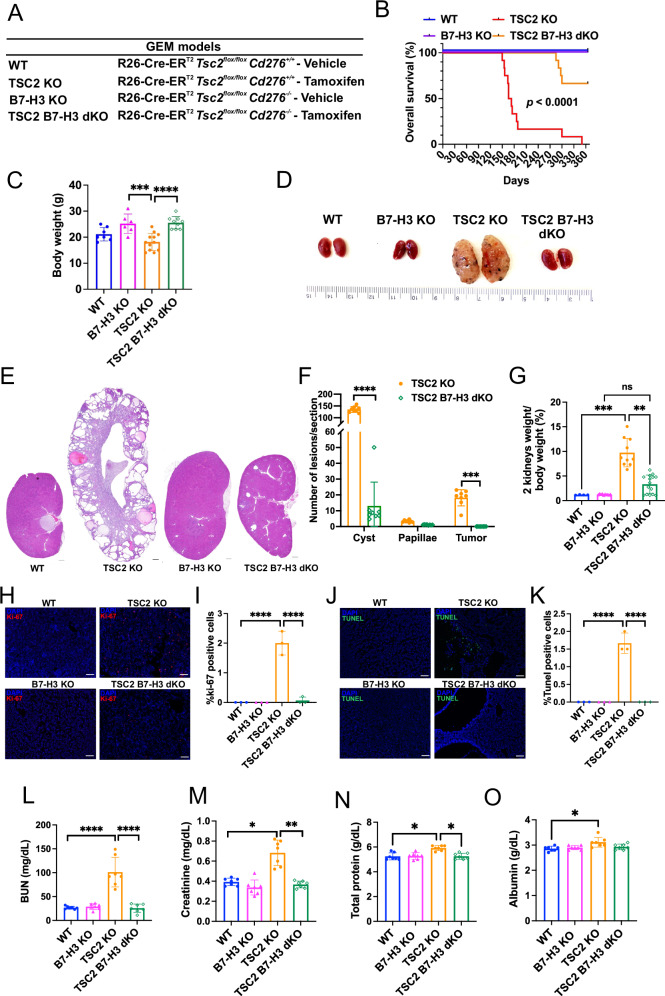
B7-H3 deletion suppresses cyst and tumor formation in TSC2 KO mice. **A** Table summarizing the transgenic mouse models and their genotypes. **B** Kaplan–Meier plot of WT (*n* = 17), TSC2 KO (*n* = 12), B7-H3 KO, (*n* = 14) and TSC2 B7-H3 dKO (*n* = 12) mice survival. **C** Quantification of the body weight in WT (*n* = 7), B7-H3 KO (*n* = 6), TSC2 KO (*n* = 12), and TSC2 B7-H3 dKO (*n* = 9) mice. Data are presented as mean ± SD, analyzed using one-way ANOVA with Tukey’s multiple comparisons test; ***p < 0.001, ****p < 0.0001. **D** Representative kidneys from 24-week-old WT, TSC2 KO, B7-H3 KO, and TSC2 B7-H3 dKO mice. **E** Representative H&E images of whole kidney sections from 24-week-old mice of the indicated genotypes. Scale bar = 1 mm. **F** Quantification of the number of kidney lesions from the indicated genotypes, obtained by counting 10 sections per kidney (*n* = 8/group). Data are presented as mean ± SD, analyzed using two-way ANOVA with Holm-Sidak multiple comparisons test;***p < 0.001, ****p < 0.0001. **G** Quantification of the kidney weight-to-body weight ratio in WT (*n* = 4), B7-H3 KO (*n* = 10), TSC2 KO (*n* = 10), and TSC2 B7-H3 dKO (*n* = 15) mice. Data are presented as mean ± SD, analyzed using one-way ANOVA with Kruskal–Wallis multiple comparisons test; **p < 0.01, ***p < 0.001. **H** Representative images of Ki-67 IF staining in sections of kidneys from mice of the indicated genotypes (*n* = 3). Scale bar = 100 µm. **I** Quantification of the Ki-67 positive cells in WT, B7-H3 KO, TSC2 KO, and TSC2 B7-H3 dKO mice (*n* = 3/group). Data are presented as mean ± SD, analyzed using one-way ANOVA with Tukey’s multiple comparisons test; ****p < 0.0001. **J** Representative images of TUNEL staining in sections of kidney from mice of the indicated genotypes. Scale bar = 100 µm. **K** Quantification of the TUNEL-positive cells in WT, B7-H3 KO, TSC2 KO, and TSC2 B7-H3 dKO mice (*n* = 3/group). Data are presented as mean ± SD, analyzed using one-way ANOVA with Tukey’s multiple comparisons test; ****p < 0.0001. Quantification of blood urea nitrogen (BUN) (**L**), creatinine (**M**), total protein (**N**), and Albumin (**O**) levels in plasma samples from WT, B7-H3 KO, TSC2 KO, and TSC2 B7-H3 dKO mice (*n* = 7/group). Results are presented as mean ± SD, analyzed using one-way ANOVA with Kruskal–Wallis multiple comparisons test; *p < 0.05, **p < 0.01, ****p < 0.0001.

Conventional ChRCC is characterized by large polygonal cells with pale, reticulated cytoplasm, distinct cell borders, and perinuclear clearing. In contrast, sarcomatoid ChRCC exhibits biphasic histology, consisting of areas of classic chromophobe morphology alongside poorly differentiated, spindle-shaped cells [2, 62]. Sarcomatoid ChRCC is notably more aggressive and is associated with significantly reduced overall survival compared to its conventional counterpart [63–65]. We used IHC to assess the expression of B7-H3 and its correlation with ChRCC hallmarks in 32 conventional and 11 sarcomatoid ChRCCs. These analyses revealed that B7-H3 is expressed in both conventional and sarcomatoid ChRCC (Fig. 4E). In conventional ChRCC, B7-H3 expression is positively correlated with *MUC20* and *HEPACAM2*, whereas in sarcomatoid ChRCC, it is primarily associated with *HEPACAM2* expression (Fig. 4F). These observations led us to hypothesize that B7-H3 may be responsible for the development of the TSC-associated ChRCC-like tumors observed in TSC2 KO mice. To test this, we generated double knockout (dKO) mice by crossing TSC2 KO mice with whole-body B7-H3 KO mice. B7-H3 KO mice alone exhibit no detectable phenotypes in any organ, have a lifespan comparable to that of wild-type mice, and do not develop kidney cysts or tumors (Fig. 5A). As shown in Fig. 1B, TSC2 KO mice exhibit a 50% survival rate at approximately 165 days, whereas all B7-H3 KO mice survive beyond 1 year. In contrast, TSC2 B7-H3 dKO mice begin to die around 285 days (Fig. 5B), likely due to kidney-specific pathology associated with *Tsc2* loss. On average, WT mice weighed 21 g, B7-H3 KO mice 25 g, TSC2 KO mice 18 g, and TSC2 B7-H3 dKO mice 25 g (Fig. 5C). B7-H3 KO mice show no overt phenotype and have a lifespan similar to WT mice (Fig. 5B) [41]. TSC2 B7-H3 dKO mice exhibited a significantly milder and delayed cystic phenotype (Fig. 5D, E), with no detectable papillary or solid tumors (Fig. 5F), and a reduced kidney-to-body weight ratio (Fig. 5G). In addition, TSC2 B7-H3 dKO mice exhibit a reduction in Ki-67-positive cystic cells and TUNEL-positive cystic cells compared with TSC2 KO mice (Fig. 5H–K). Furthermore, TSC2 B7-H3 dKO mice exhibit improved renal function, reaching levels comparable to those of WT mice (Fig. 5L, M). TSC2 KO mice exhibit increased serum total protein levels, particularly albumin, compared with WT mice; these levels are restored to those observed in WT mice in TSC2 B7-H3 dKO mice (Fig. 5N, O).

We collected kidneys from 22-week-old TSC2 KO and TSC2 B7-H3 double knockout (dKO) mice (*n* = 3 per group) and performed scRNA-seq to assess the impact of B7-H3 loss on the transcriptomic landscape of TSC2-deficient kidneys. Quality control measures were applied during data processing to exclude doublets, and RPCA-based integration was used to correct for batch effects (Supplementary Fig. 5A, B). This yielded a total of 42,894 high-quality single-cell transcriptomes, 23,162 from TSC2 KO and 19,732 from TSC2 B7-H3 dKO mice (Supplementary Fig. 5C). Cells were subsequently classified into 14 major cell types based on canonical cell-defining marker genes (Supplementary Fig. 5D). We observed a 60% reduction (*P* = 0.062) in the proportion of cyst/tumor cells in TSC2 B7-H3 dKO kidneys compared to TSC2 KO controls (Supplementary Fig. 5E). As expected, there is a proportional increase in proximal tubule cells, rising from 28% in TSC2 KO to 55% in TSC2 B7-H3 dKO kidneys (Supplementary Fig. 5E). Additionally, we noted an increased proportion of artery/vein endothelial cells (Artery/Vein) and a reduction in macrophage (Mac) populations in the TSC2 B7-H3 dKO group (Supplementary Fig. 5E). Differential gene expression analysis in cyst/tumor cells revealed only a single downregulated gene in TSC2 B7-H3 dKO compared to TSC2 KO mice (Supplementary Fig. 5F). Transcriptomic differences across other cell types were minimal (Supplementary Fig. 5F), suggesting that B7-H3 deletion has a limited influence on the global transcriptional profile of TSC2-deficient kidneys.

### Loss of B7-H3 attenuates Tsc2 deficiency-driven enhancement of amino acid and aminoacyl-tRNA biosynthesis

**Fig. 6:**
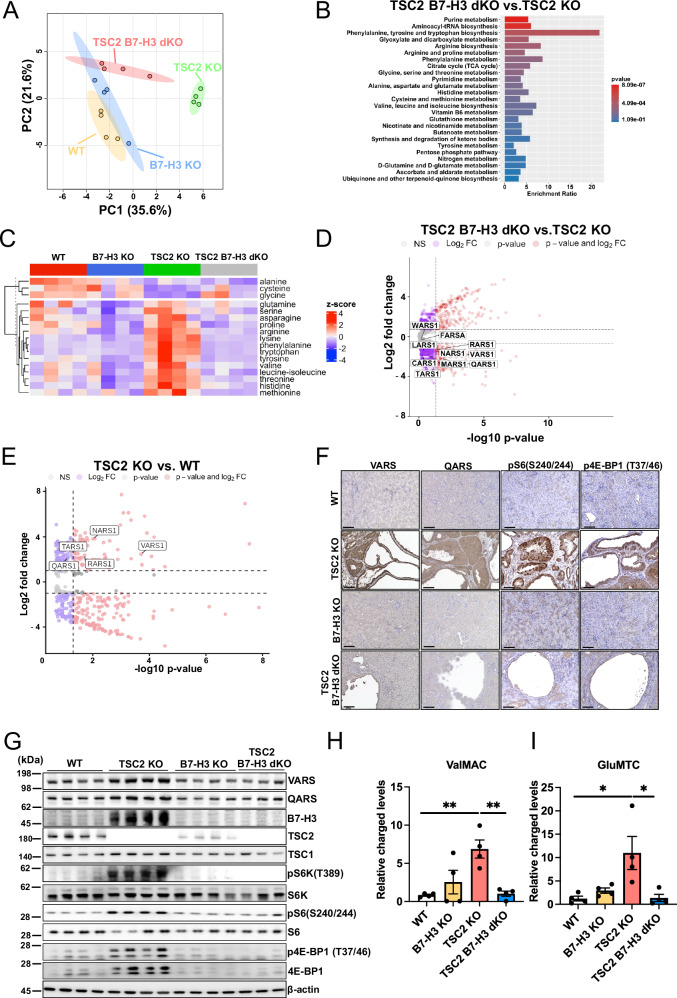
B7-H3 deficiency attenuates the TSC2 loss-induced upregulation of amino acid and aminoacyl-tRNA biosynthesis. **A** Principal component analysis (PCA) of metabolomic profiles from mice of the indicated genotypes (*n* = 4/group). The percentages indicate the explained variance for PC1 and PC2. **B** Metabolite Set Enrichment Analysis (MSEA) presents the top 25 related metabolic pathways, ranked by *p* value. **C** Heatmap showing z-score normalized metabolite abundance profiles in renal tissues from mice of the indicated genotypes. Volcano plot showing differentially expressed proteins in the kidneys of TSC2 B7-H3 dKO versus TSC2 KO mice (**D**) and TSC2 KO versus WT mice (**E**). Each dot represents a protein. Proteins with absolute log_2_ fold-change of 1.5 and adjusted *p* value *p* ≤ 0.05 are represented in red. **F** Representative images of VARS, QARS, pS6(S240/244), and p4E‑BP1(T37/46) IHC staining in sections of kidney from mice of the indicated genotypes (*n* = 5/group). Scale bar = 100 µm. **G** Immunoblot analysis of kidney tissue lysates from WT (*n* = 4), TSC2 KO (*n* = 4), B7-H3 KO (*n* = 4) and TSC2 B7-H3 dKO (*n* = 3) mice. Quantification of charging levels of tRNA^ValMAC^ (**H**) and tRNA^GluMAC^ (**I**) in kidneys from indicated genotypes of mice (*n* = 4/group). Results are presented as mean ± SD, analyzed using one-way ANOVA with Tukey’s multiple comparisons test; *p < 0.05, **p < 0.01.

To investigate how B7-H3 ablation mitigates cystic and tumor phenotypes in TSC2 KO kidneys, we conducted integrated metabolomic and proteomic profiling of kidneys from WT, TSC2 KO, B7-H3 KO, and TSC2 B7-H3 dKO mice. A targeted metabolomics approach profiling 300 polar metabolites using mass spectrometry revealed profound metabolic alterations in TSC2 KO kidneys compared to WT (Fig. 6A). In contrast, B7-H3 KO kidneys display a metabolic profile largely similar to WT (Fig. 6A). Strikingly, the metabolic profile of TSC2 B7-H3 dKO kidneys more closely resembled that of B7-H3 KO and WT mice (Fig. 6A). Metabolite Set Enrichment Analysis (MSEA) comparing TSC2 B7-H3 dKO and TSC2 KO kidneys identified purine metabolism as the most significantly downregulated pathway, followed by aminoacyl-tRNA biosynthesis (Fig. 6B). TSC2 loss led to increased abundance of various amino acids, including lysine, phenylalanine, tryptophan, tyrosine, valine, leucine-isoleucine, threonine, histidine, and methionine, all of which were markedly reduced upon B7-H3 loss in the TSC2 KO kidneys (Fig. 6C).

Global proteomic profiling revealed downregulation of multiple aminoacyl-tRNA synthetases, including VARS1 (valyl-tRNA synthetase), QARS1 (glutaminyl-tRNA synthetase), NARS1 (asparaginyl-tRNA synthetase), RARS1 (arginyl-tRNA synthetase), TARS1 (threonyl-tRNA synthetase), and MARS1 (methionyl-tRNA synthetase) in TSC2 B7-H3 dKO compared with TSC2 KO mice (Fig. 6D). Interestingly, TSC2 KO also led to an increase in several aminoacyl-tRNA biosynthesis proteins, including VARS1, NARS1, RARS1, TARS1, and QARS1, compared with WT mice (Fig. 6E). These enzymes catalyze the esterification of specific amino acids to their cognate tRNAs, a critical step in translation. This proteomic signature is consistent with our metabolic data, reinforcing that aminoacyl-tRNA biosynthesis is among the most significantly suppressed pathways upon B7-H3 deletion in the TSC2 KO background. Indeed, immunohistochemistry and immunoblotting confirmed elevated protein levels of VARS1 and QARS1 in TSC2 KO kidneys, which were reduced in TSC2 B7-H3 dKO tissues (Fig. 6G). Furthermore, tRNA charging assays reveal increased charging of tRNA^ValMAC^ and tRNA^GluMAC^ in TSC2 KO kidneys, both of which were attenuated in the absence of B7-H3 (Fig. 6H, I). Collectively, these data suggest that B7-H3 contributes to TSC2-loss-driven kidney pathology, at least in part, through the upregulation of amino acid metabolism and aminoacyl-tRNA biosynthesis, implicating translational control as a key downstream effect of B7-H3 in this context.

### NRF1-B7-H3 axis regulates proteasome pathways in TSC2 KO kidneys

**Fig. 7:**
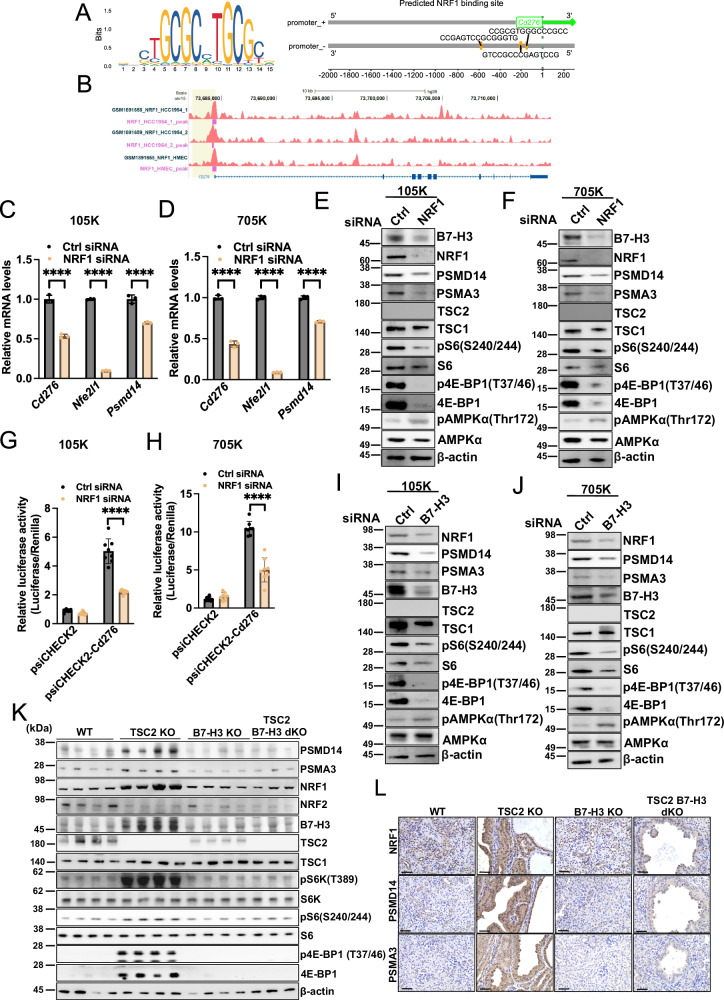
NRF1 transcriptionally induces the expression of B7-H3 and proteasome genes in TSC2 KO Kidneys. **A** The NRF1-binding motif predicted by JASPAR, and schematic images showing the potential NRF1 binding sites in the B7-H3 promoter region (Score > 6). **B** UCSC Genome Browser showing binding peaks of NRF1 in the B7-H3 promoter region (GSE67867). siRNA knockdown of NRF1 reduces the mRNA expression of *Cd276*, *Nfe2l1*, and *Pmsd14* in *Tsc2*-deficient 105K cells (**C**) (*n* = 3) and 705K cells (**D**) (*n* = 3). Results are presented as mean ± SD, analyzed using two-way ANOVA with Sidak’s multiple comparisons test; ****p < 0.0001. siRNA knockdown of NRF1 reduces B7-H3 protein expression in *Tsc2*-deficient 105K cells (**E**) (*n* = 3) and 705K cells (**F**) (*n* = 3). *Cd276* promoter activity is suppressed by NRF1 knockdown in *Tsc2*-deficient 105K cells (**G**) (*n* = 7-8) and 705K cells (**H**) (*n* = 8). Results are presented as mean ± SD, analyzed using two-way ANOVA with Sidak’s multiple comparisons test; ****p < 0.0001. siRNA knockdown of B7-H3 reduces NRF1 protein expression in *Tsc2*-deficient 105K cells (**I**) (*n* = 3) and 705K cells (**J**) (*n* = 3). **K** Immunoblot analysis of whole-kidney tissue lysates from WT (*n* = 4), TSC2 KO (*n* = 4), B7-H3 KO (*n* = 4), and TSC2 B7-H3 dKO (*n* = 3) mice. **L** Representative images of NRF1, PSMD14 and PSMA3 IHC staining in kidney sections from mice of the indicated genotypes (*n* = 5/group). Scale bar = 100 µm.

As noted above, B7-H3/*CD276* mRNA expression is elevated in TSC2 KO kidneys (Fig. 4A–C). To identify the transcription factors regulating B7-H3/*CD276*, we analyzed its promoter region using JASPAR [66] and identified NRF1 as a candidate binding factor (Fig. 7A). Consistently, ENCODE NRF1 ChIP-seq data revealed significantly enriched NRF1-binding sites in the B7-H3/*CD276* promoter region (Fig. 7B). NRF1 knockdown reduced both B7-H3 mRNA and protein expression (Fig. 7C–F) and suppressed CD276 promoter activity by more than 50% in *Tsc2*-deficient 105K and 705K cell lines (*P* < 0.0001) (Fig. 7G, H). Interestingly, B7-H3 knockdown also diminished NRF1 protein expression, suggesting a potential regulatory feedback loop (Fig. 7I, J). Furthermore, proteasome subunits, including PSMD14 and PSMA3, are reduced following B7-H3 silencing (Fig. 7I, J). We also observed a decrease in phospho-S6^S240,244^ levels following NRF1 or B7-H3 knockdown (Fig. 7E, F, I, L). AMPK is an upstream regulator of the NRF1–proteasome axis, and we found that the levels of phosphao-AMPKα^Thr172^ is increased, suggesting that AMPK is activated following NRF1 or B7-H3 knockdown (Fig. 7E, F, I, L). Immunoblotting and immunohistochemistry demonstrated that TSC2 deficiency enhanced NRF1, but not NRF2, protein expression in renal tissue, an effect reversed by B7-H3 deletion (Fig. 7K, L) RNA-seq and global proteomic analyses further showed upregulation of multiple proteasome subunits, including PSMD14 and PSMA3 in TSC2 KO kidneys compared with WT controls, whereas loss of B7-H3 markedly attenuated this upregulation (Supplementary Fig. 7A, B). Notably, at the early 3-month time point, TSC2 B7-H3 dKO mice exhibit a phenotype similar to that of 3-month-old TSC2 knockout mice (Supplementary Fig. 6A). However, phospho-S6^S235,236^ levels are decreased (Supplementary Fig. 6B, C), suggesting that B7-H3 regulates *Tsc2* loss-driven cystic and tumor formation by decreasing mTORC1 activity. Collectively, these findings demonstrate that the NRF1-B7-H3 axis sustains proteasome pathway activation, thereby contributing to the pathological progression of TSC2-deficient kidneys.

## Discussion

In this study, we demonstrate that conditional embryonic deletion of *Tsc2* in mice results in the formation of kidney cysts and solid tumors that resemble human ChRCC. This phenotype is similiar to that observed in a previously reported *Flcn*^+/−^ mouse model of Birt-Hogg-Dube syndrome which also develops oncocytic and chromophobe renal tumors, although with lower levels of phospho-ribosomal protein S6 in the tumor cells compared with normal kidney [16]. Notably, single-cell transcriptomic analysis of kidneys from TSC2 KO mice revealed a novel cyst/tumor cell population enriched for intercalated cell markers and characterized by high mTORC1 signaling in the Foxi1^+^Rhcg^+^ cell cluster. Sub-clustering identified ChRCC-like transcriptional programs and pathway activities, including FOXI1, HEPACAM2, and MUC20 expression. Notably, B7-H3 is highly expressed in these tumors, and its genetic ablation markedly suppressed cyst and tumor formation. Multi-omics analysis further identified defects in amino acid metabolism and aminoacyl-tRNA biosynthesis in the TSC2 B7-H3 dKO mice, suggesting that B7-H3 is required for TSC2-driven tumorigenesis. These results highlight the key role of B7-H3 in TSC2-driven ChRCC and a potential target for therapeutic intervention. Interestingly, B7-H3 has recently been found to be highly expressed in another rare subtype of RCC, translocation RCC [67].

Type A intercalated cells are widely considered the cells of origin for ChRCC, renal oncocytoma (RO) tumors, and low-grade oncocytic tumors (LOT) [68, 69]. Prior studies have defined c-KIT, the ammonium transporters Rhcg and Rhbg, and the transcription factor Foxi1 as the markers of intercalated cells [68, 70]. Here, we discovered a population of cyst and tumor cells that exhibit molecular characteristics of intercalated cells by expressing Rhcg and Foxi1 and exhibit elevated mTORC1 signaling in the TSC2 KO mice, supporting the fact that ChRCC arises from type A intercalated cells. We also acknowledge that, because the current model is a tamoxifen-inducible conditional knockout, variability in the cell types undergoing *Tsc2* deletion represents an inherent limitation. Future studies incorporating Rosa26 reporter mice with lineage tracing and more precise assessment of the spatial and temporal patterns of Cre-mediated recombination will be needed to address this. Interestingly, within the cyst/tumor cell population, we observed a sub-cluster of cells that co-expressed markers of both intercalated (Rhcg) and principal (Hsd11b2) cells, implying that these cells may derive from a transitional cell state (Fig. 3D). Transitional cells within the collecting duct have previously been described and are thought to emerge in response to environmental acid/base cues [71]. Our findings suggest that loss of *Tsc2* may drive the expansion of these collecting duct transitional cells. Moreover, previous studies have shown that TSC2 and Rheb regulate Notch-dependent cell-fate decisions in Drosophila [72, 73]. Whether Notch signaling similarly regulates the expansion of kidney ductal transitional cells remains to be determined. We found that the Rhcg^+^Foxi1^+^ cyst/tumor cells displayed a stronger mTORC1 signature compared with other cyst/tumor cells. Interestingly, recent studies indicate that a prominent mTORC1 signature is typically associated with dedifferentiated ChRCC, raising the possibility that Rhcg⁺Foxi1⁺ tumor cells represent a more aggressive, metastasis-prone population within ChRCC [65].

B7-H3 upregulation exerts a significant inhibitory immune effect within the tumor microenvironment, particularly impacting T cell subsets. Recent studies have demonstrated that tumor-expressed B7-H3 inhibits the function of CD8^+^ T cells [40]. Additionally, B7-H3 inhibition has been associated with increased cytotoxic CD38^+^CD39^+^CD4^+^ T cells [31] in a syngeneic mouse model of *Tsc2*-deficient 105K tumors. Aberrant B7-H3 expression has been documented in clear cell renal cell carcinoma (ccRCC) [74], where it is present in both tumor cells and vasculature and serves as an independent predictor of poor prognosis, correlating with PD-L1 expression and increased regulatory T cell infiltration [74, 75]. However, the expression patterns and regulatory mechanisms of B7-H3 in ChRCC remain largely undefined. Here, we show that B7-H3 is expressed in both conventional ChRCC and sarcomatoid ChRCC (Fig. 4E). Consistent with these findings, B7-H3 was also strongly expressed in the cysts and tumors of TSC2 KO mice (Fig. 3A, B). Genetic ablation of B7-H3 delayed cyst formation and improved renal function in TSC2 KO mice (Fig. 5A–G, L–O). Unexpectedly, our scRNA-seq analysis revealed that loss of B7-H3 did not alter the transcriptional profile of cyst/tumor cells (Supplementary Fig. 5F), suggesting that B7-H3 regulates kidney cyst/tumor development through mechanisms independent of transcriptional reprogramming. Furthermore, the impact of B7-H3 ablation on kidney T cells was minimal (Supplementary Fig. 5F), indicating that B7-H3 promotes kidney tumorigenesis through a T cell-independent pathway.

Metabolic reprogramming is critical to the pathogenesis of ChRCC [2]. ChRCC is characterized by reduced nutrient homeostasis and increased abundance of the proteasome, ubiquitin-mediated proteolysis, and lysosomes [76]. Additionally, reprogramming of glutathione homeostasis is a signature feature of ChRCC. ChRCC cells are highly dependent on cysteine/cystine uptake to maintain high glutathione (GSH) levels, which are regulated by the expression of Gamma-glutamyl transferase 1 (GGT1) [6, 7]. Consistent with these results, we found the levels of various amino acids, including glutamine, had increased in the TSC2 KO model (Fig. 6C). Proteomic signature further indicates multiple aminoacyl-tRNA synthetases are increased with metabolic alterations (Fig. 6D), suggesting TSC2 loss may affect the tumorigenesis of ChRCC through regulating aminoacyl-tRNA-dependent protein translation. We also found that the metabolic profile of kidneys from TSC2 KO mice differs from prior reported patterns in patients with ChRCC [76]. This difference may be attributed to distinct underlying amino acid response mechanisms. ChRCC cells could preferentially internalize and catabolize external macromolecules as a source of amino acids [76], while TSC2 works in concert with Rag GTPase to regulate mTORC1 activity in response to both amino acid and growth factor restriction, suggesting TSC may drive distinct metabolic reprogramming processes involved in the mechanism of ChRCC [77–79]. Interestingly, B7-H3 inhibition reduced the abundance of amino acids, and global proteomic profiling revealed downregulation of multiple aminoacyl-tRNA synthetases, particularly VARS1 and QARS1 (Fig. 6B–D). The availability of amino acids is crucial for translation control [80]. These data imply that B7-H3 may work as an upstream effector of protein synthesis and degradation.

mTORC1 signaling pathway plays an essential role in regulating mRNA translation and protein synthesis [81]. Previous studies demonstrated that TSC2 loss-driven mTORC1 activation elevates proteasome levels in an NRF1-dependent manner to increase the intracellular amino acid pool and new protein synthesis rates [82]. Consistent with these results, we found TSC2 loss enhanced the protein expression of the transcription factor NRF1 and multiple proteasome subunits (Fig. 7K and Supplementary Fig. 7A, B). This response was reversed upon B7-H3 deletion, suggesting that B7-H3 may be involved in regulating protein synthesis. This hypothesis was further confirmed in TSC2-deficient 105K and 705K cell lines through NRF1 knockdown (Fig. 7E, F). Our previous studies demonstrated that B7-H3 is regulated by transcription factor YY2, which binds to the B7-H3 promoter [31]. NRF1 is also predicted to bind the B7-H3 promoter region (Fig. 7A, B). Our experimental results demonstrate that NRF1 binds to the B7-H3 gene promoter (Fig. 7G, H). Loss of NRF1 downregulates *CD276* transcription in cells with hyperactive mTORC1 (Fig. 7C, D). Together, these findings suggest that B7-H3 loss downregulates the NRF1-dependent proteasome biogenesis in TSC2-deficient kidneys, thereby alleviating the tumor phenotype.

In conclusion, we developed a novel mouse model of TSC-associated ChRCC via conditional *Tsc2* deletion. Single-cell transcriptomics identified a distinct type A intercalated cell-derived tumor population with hyperactive mTORC1 signaling. Importantly, B7-H3 ablation suppresses TSC2-deficient tumorigenesis by disrupting amino acid metabolism and aminoacyl-tRNA biosynthesis, highlighting B7-H3 as a potential therapeutic target and providing a mechanistic basis for precision interventions in TSC-associated renal tumors.

## Supplementary information

Supplemental Material

## Data Availability

The scRNA-seq data are openly available in Gene Expression Omnibus (GEO) at GSE307301.
