# Supplementary material for: mTORC1 activation induces B7-H3-dependent metabolic reprogramming in renal cell carcinoma (RCC)
Source: Oncogene. 2026 Jul 20;45(34):3525–40. doi: 10.1038/s41388-026-03891-w (PMC13481258; doi:10.1038/s41388-026-03891-w)
Supplement: Supplementary file 1 [file 41388_2026_3891_MOESM1_ESM.pdf]

Supplementary Information

mTORC1 activation induces B7-H3–dependent metabolic reprogramming in renal cell carcinoma

Keying Li[1,#], Heng Du[3,4,#], Yifan Wang[1], Damir Khabibullin[5], Michel Alchoueiry[5], Melissa Daou[5], John M. Asara[6,7], David J. Kwiatkowski[5], Elizabeth P. Henske[5,*], Heng-Jia Liu[1,2,*]

This PDF file includes:

Supplementary Methods

Supplementary Figures 1-7

Supplementary Tables 1-2

Supplementary References

Supplementary Methods

Single-cell RNA sequencing

Kidneys were dissociated using a modified version of a published protocol described in ref [1]. The kidneys were minced and transferred into a pre-cooled Miltenyi C-tube containing a protease solution (5 mM $CaCl_2$ [Invitrogen, Cat#AM9530G], 10 mg/mL *B. Licheniformis* protease [Sigma-Aldrich, Cat#P5380], 125 U/mL DNase I [Sigma-Aldrich, Cat#D5025], 1xPBS), then incubated with gentle rotation at 4 ^oC for 10 min. After incubation, the samples were run on Miltenyi gentleMACS brain_03 program for three times in a cold room (4 ^oC). The incubation was repeated with gentle rotation at 4 ^oC for 10 min. Single-cell dissociation was confirmed by microscopic examination followed by the addition of 3mL ice-cold PBS with 10% FBS. Cells were passed through a 70 μm strainer and filter was rinsed with 3 mL ice-cold PBS with 10%FBS. Then cells were centrifuged at 300 x *g* at 4 ^oC for 5 min and passed through 40 μm strainer for two times. Dead cells were removed using Dead Cell Removal MicroBeads (Miltenyi, Cat#130-090-101), then excess red blood cells were removed using Anti-Ter-119 MicroBeads (Miltenyi, Cat# 130-049-901). Cells were resuspended in PBS with 0.04% BSA at 1000 cells/mL. Single-cell suspensions were super-loaded onto a single lane (Chromium chip G, 10X Genomics) followed by cDNA and library generation according to the manufacturer's protocol. mRNA libraries were sequenced to an average of 50,000 reads per cell using Illumina Novaseq.

Quality control and filtering of single-cell RNA sequencing data

Raw data were processed by CellRanger (v6.1.1) for sample demultiplexing, barcode processing, and UMI counting, with alignment to the mm10 reference mouse genome. The gene-barcode matrix was then conducted in R (v4.2.2) following Seurat (v5.0.0) package. Quality control was first performed independently to remove low-quality cells that have less than 300 unique feature counts, no higher than 50 gene numbers, more than 35% mitochondrial counts. The data was then normalized, log transformed and scaled.

Feature selection, data integration and cell-type annotation

Data were integrated by the Seurat's reciprocal PCA (RPCA) fast integration pipeline. Principal component analysis was performed and uniform manifold approximation and projection (UMAP) or t-distributed neighbor embedding (tSNE) was used to classify cells into different cell clusters with resolution=0.5. To define the significance of gene expression changes in cell clusters, we used function FindMarkers with "wilcox" test method. To annotate cell type, we used clustifyr [2] to annotate cell types based on the gene expression profile from a prior publication[3].

Differentially expressed genes analysis

We used DESeq2 package for analysis of aggregated read counts [4]. We first aggregated reads across biological triplicates of all cells from WT, TSC2 KO, B7-H3 KO and TSC2 B7-H3 dKO kidneys, transforming a genes-by-cells matrix to a genes-by-triplicates matrix using matrix multiplication. Then we used a Wald test of negative binomial model coefficient and likelihood ratio test compared to a reduced model to

compute the statistical significance. KEGG and GSEA were performed with clusterProfiler package [5,6], which supports statistical analysis and visualization of functional profiles for gene clusters.

Calculation of enrichment score

UCell [7] was used to compute enrichment scores for each cell based on the relative ranking of genes within predefined gene sets. This approach was employed to identify cells enriched for mTORC1 signaling or ChRCC signatures within cyst/tumor clusters derived from TSC2 KO mice. The mTORC1 gene signature was obtained from the GSEA Hallmark mTORC1 signaling gene set, and chromophobe RCC marker genes were curated from Lindgren et al [8]. Based on the distribution of mTORC1 scores, cell clusters were stratified into high, medium, and low activity groups.

Transcription factor regulon analysis

DoRothEA (v1.8.1) [9] was used to infer the TF activity, and all parameters were set to default values. The viper score of TF in cell clusters was estimated by DoRothEA within the database containing interactions with confidence level A, B and C.

Pathway activity score analysis

Pathway activity scores were calculated using the progeny function (z_scores=TRUE, organism = “mouse”, top=300, perm=100) from PROGENy (v1.18.0) [10].

Cell similarity inference

Glmnet (v4.1.10) [11] was used to quantify the cell similarity between TSC2 KO and WT epithelial cells. This approach was implemented through a one-vs-rest elastic-net logistic regression framework, with transcriptomic profiles of epithelial cells from WT mice serving as the training set. For each reference cell type, a binary classifier was trained using the elastic-net penalty ($\alpha = 0.92$) and 10-fold cross-validation to select the optimal regularization parameter (λ). Each classifier predicted log-odds for all query cells and transformed to probabilities. The predicted cell type for each query cell was assigned as the class with the highest probability. Cluster-level similarities were computed as the mean probability for visualization.

Pseudo-time analysis

Slingshot (v2.14.0) [12] was used to perform a temporally ordered developmental trajectory analysis. The target cell populations were extracted and re-embedded in t-SNE space, after which slingshot was applied. The resulting curve represents the progression of cell states over pseudo-time. Pseudotime trajectory analysis of kidney epithelial cells in TSC2 KO mice was performed using Monocle3 (v1.4.26) [13], based on the learned principal graph structure in a UMAP-reduced-dimensional space.

RNA sequencing and data processing

Total RNA isolated from kidney tissues were extracted using the RNeasy Plus Micro Kit (Qiagen, Cat#74034) according to the manufacturer’s instructions. RNA integrity was verified by Agilent 2100 Bioanalyzer. mRNA was enriched via oligo (dT) magnetic

bead selection, and subsequently used to make cDNA library using the KAPA mRNA HyperPrep Kit (Roche) for sequencing on an Illumina NextSeq500 instrument (Illumina). Raw paired-end reads were aligned to the mm10 genome using STAR (v2.5). HTSeq (v0.6.1) was used to count the read numbers mapped to each gene, followed by normalization of expression levels calculated as fragments per kilobase of exon per million mapped reads (FPKM). Differential expression analysis was performed using DESeq2 R package (2_1.6.3), with significance thresholds set at |log2fold change| ≥ 1.0 and adjusted p-value < 0.05 (Benjamini-Hochberg correction). We used the ggplot2 R package (v3.4.2) to generate a Volcano plot and Heatmap.

Dual luciferase Assays

105K and 705K cells with control siRNA or NRF1 siRNA were plated in 96-well plates and transfected with 250 ng dual luciferase plasmid psiCHECK2 (Promega, Cat#C8021) or psiCHECK2 plasmid containing Cd276 promoter for 48 h. Luciferase activities were evaluated using the Dual-Glo Luciferase Reporter Assay System (Promega, #E2920). Normalized activity was calculated as Firefly / Renilla luciferase activity.

Cell culture

105K mouse kidney cyst–adenocarcinoma cell were derived and maintained as previously described [14]. 705K mouse kidney tumor cells were derived from a kidney tumor of a 12-month-old female Rosa26-CreERT2 *Tsc2*$^{flox/flox}$. All cells were cultured in DMEM supplemented with 10% FBS and 1% penicillin/streptomycin.

siRNA transfections

For RNA interference experiments, the following siRNAs (Horizon Discovery SMARTpools ON-TARGET plus) were transfected at 12.5 nM: B7-H3 (L-064148-01-0005), NRF1 (L-041037-01-0005) or control (non-targeting siRNA pool D-001810-10-05) using Lipofectamine RNAiMAX (ThermoFisher Scientific, Cat#13778150) according to manufacturer's instructions.

RNA extraction and quantitative real-time PCR

Total RNA was extracted from cells or renal tissue using the RNeasy Mini Kit (Qiagen), following manufacture's instructions. Extracted total RNA was reverse transcribed into cDNA using High-Capacity cDNA Reverse Transcription kit (ThermoFisher Scientific, Cat#4368814). Quantitative real-time PCR (qPCR) was performed using Taqman Fast Advanced Master Mix (ThermoFisher Scientific, Cat#4444963). Real-time PCR was performed using an Applied Biosystems StepONE Plus Real-time PCR system (ThermoFisher Scientific) with gene-specific primers (Table S2). Values represent the average of three independent experiments normalized to β-actin.

Immunoblotting

Immunoblotting was performed as described previously[15]. Briefly, cells and tissues were lysed in RIPA buffer (CST, Cat#9806) with protease inhibitor cocktail (Sigma, Cat#P8340) and phosphatase inhibitor cocktail (Sigma, Cat# P0044). Total cell lysates

were rotated at 4 °C for 30 min and centrifuged at 4 °C for 10 min. Total protein was separated via 4–12% SDS-PAGE and transferred to PVDF membranes. The membranes were blocked with TBS containing 0.1% Tween-20 and 5% bovine serum albumin and then incubated with primary antibodies overnight at 4 °C. After incubation with the appropriate horseradish peroxidase-conjugated secondary antibodies, the membranes were developed using SuperSignal™ West Pico PLUS Chemiluminescent Substrate (ThermoFisher Scientific, Cat#34580) and the images were captured using iBright Western Blot Imaging system (ThermoFisher Scientific). The following primary antibodies were used for immunoblotting at 1:1000 dilution unless otherwise indicated: Phospho-p70 S6 Kinase T389 (CST, Cat#9234), p70 S6 Kinase (CST, Cat#2708), TSC1 (Abcam, Cat# ab270967), TSC2 (CST, Cat#4308), pS6 S240/244 (1:600, CST, Cat# 5364), S6 (CST, Cat#2217), p4E-BP1 T37/46 (CST, Cat#2855), 4E-BP1 (CST, Cat#9644), mouse B7-H3 (1:500, R&D Systems, Cat#AF1397), Vinculin (CST, Cat#4650), VARS (GeneTex, Cat#GTX66016), QARS (GeneTex, Cat#GTX33449), PSMD14 (CST, Cat#4197), PSMA3 (CST, Cat#12446), NRF1/TCF11 (CST, Cat#8052), NRF2 (CST, Cat#20733), pAMPKα Thr172 (CST, Cat#2535), AMPKα (CST, Cat#2532), β-actin (Sigma-Aldrich, Cat#A978).

Biochemical examination of serum creatinine and BUN

Approximately 400–500 µL of blood was collected from each mouse into gold-top serum separator tubes and allowed to clot at room temperature for 30 minutes. Samples were centrifuged at 2000 × g for 10 minutes, and the serum was carefully transferred to clean tubes. Serum samples were submitted to VRL Animal Health Diagnostics for renal function analysis, including measurements of creatinine and blood urea nitrogen (BUN) levels.

TUNEL Staining

Frozen sections were fixed with 4% paraformaldehyde, permeabilized with proteinase K, and incubated with the TUNEL reaction mixture for 60 minutes at 37°C. Sections were counterstained with DAPI to visualize nuclei. Representative images were taken using a Keyence fluorescence microscope and quantified by counting the positive cells of five different fields using Fiji ImageJ.

Immunohistochemistry and Immunofluorescence

Immunohistochemistry was performed as described previously[15]. Briefly, Tissues were dewaxed in 3 changes of xylene followed by 3 changes of 100% ethanol. Heat-induced antigen retrieval was performed in a Russell Hobbs pressure cooker for 10 min in sodium citrate antigen retrieval solution (10 mM sodium citrate, pH 6.0). Sections were blocked in 5% normal goat serum for 60 min, followed by incubation with B7-H3 (1:200, CST, Cat#14058), Phospho-S6 S235/236 (CST, Cat# 2211), vimentin (1:100, CST, Cat#5741), VARS (1:300, GeneTex, Cat#GTX66016), QARS (1:300, GeneTex, Cat#GTX33449), NRF1/TCF11 (1:200, CST, Cat#8052), PSMD14 (1:250, CST, Cat#4197), PSMA3 (1:75, CST, Cat#12446), FOXI1 (1:200, Atlas Antibodies, Cat#HPA071469), HEPACAM2 (1:200, LSBio, Cat# LS-B8888), MUC20 (1:200, Mybiosource, Cat#MBS1752535) antibodies at 4° C overnight. Sections were incubated with polymer anti-rabbit IgG (Vector Laboratories, Cat#MP-7451) at RT for 30 min. Signal

was detected using DAB substrate, Peroxidase (Vector Laboratories, Cat#SK-4105) according to manufacturer's instructions. Slides were counterstained with hematoxylin (Agilent, Cat#S330930-2) and mounted with D.P.X. Representative images were taken using a Keyence fluorescence microscope and quantified by counting the positive cells of five different fields/tumor using Fiji ImageJ.

Frozen tissue sections were fixed with cold methanol for 20 min. Sections were blocked and stained with B7-H3 (1:250, Abcam, Cat#ab134161), vimentin (1:100, CST, Cat#5741), Phospho-S6 S235/236 (CST, Cat# 2211), Ki-67 (1:200, thermo, Cat#14-5698-80), Cre (1:200, CST, Cat#15036T), TSC2 (1:200, CST, Cat#4308) and LRP2 (1:200, Santa Cruz, Cat#SC-515772). Sections were incubated with polymer anti-rabbit IgG (Vector Laboratories, Cat#MP-7451) at RT for 30 min. Signal was detected using DAB substrate, Peroxidase (Vector Laboratories, Cat#SK-4105) according to manufacturer's instructions. For immunofluorescence, sections were incubated with Alexa Fluor® 488 Goat Anti-Mouse IgG (thermo , Cat# A-11001) and Alexa Fluor® 594 Goat Anti-Rabbit IgG (thermo, Cat#A-11012) at RT for 2h. Slides were counterstained with hematoxylin (Agilent, Cat#S330930-2) or DAPI (Beyotime, Cat#C1002), mounted with D.P.X., imaged, and quantified as described above.

Targeted Metabolite Mass Spectrometry

Metabolites from 20mg of grounded renal tissues were extracted with 88% HPLC grade methanol and 12% PBS. Samples were re-suspended using 20 uL HPLC grade water for mass spectrometry. 5-7 µL were injected and analyzed using a hybrid 6500 QTRAP triple quadrupole mass spectrometer (AB/SCIEX) coupled to a Prominence UFLC HPLC system (Shimadzu) via selected reaction monitoring (SRM) of a total of 300 endogenous water soluble metabolites for steady-state analyses of samples. Some metabolites were targeted in both positive and negative ion mode for a total of 311 SRM transitions using positive/negative ion polarity switching. ESI voltage was +4950V in positive ion mode and –4500V in negative ion mode. The dwell time was 3 ms per SRM transition and the total cycle time was 1.55 seconds. Approximately 9-12 data points were acquired per detected metabolite. Samples were delivered to the mass spectrometer via hydrophilic interaction chromatography (HILIC) using a 4.6 mm i.d x 10 cm Amide XBridge column (Waters) at 400 µL/min. Gradients were run starting from 85% buffer B (HPLC grade acetonitrile) to 42% B from 0-5 minutes; 42% B to 0% B from 5-16minutes; 0% B was held from 16-24 minutes; 0% B to 85% B from 24-25 minutes; 85% B was held for 7 minutes to re-equilibrate the column. Buffer A was comprised of 20 mM ammonium hydroxide/20 mM ammonium acetate (pH=9.0) in 95:5 water:acetonitrile. Peak areas from the total ion current for each metabolite SRM transition were integrated using MultiQuant v3.0.2 software (AB/SCIEX).

Global proteomics

Protein pellet from 20mg of grounded kidney tissues were extracted with 88% HPLC grade methanol and 12% PBS by centrifugation at 500g at 4°C. Then, the samples were analyzed by positive-ion mode LC-MS/MS using a high-resolution hybrid QExactive HF Orbitrap Mass Spectrometer (Thermo Fisher Scientific) via higher-energy collisional dissociation with data-dependent analysis with 1 MS1 scan followed by 8 MS2 scans per cycle for the top 8 ions detected in the MS1 scan. Results were imported into Scaffold

Q+S 4.6 software (Proteome Software Inc.) with a peptide threshold of about 85% and a protein threshold of 95%, resulting in a peptide false discovery rate of approximately 1%. Further statistical analysis was performed using PANTHER (http://www.pantherdb.org/).

tRNA charging assays

Kidney tissues were homogenized in liquid nitrogen and lysed with cold TRIzol. Lysates were shaken with chloroform, centrifuged and precipitated with cold ethanol in presence of GlycoBlue Coprecipitant overnight. Samples were resuspended in tRNA reprecipitation buffer with cold EtOH, and precipitated overnight again. The following day, 2 µg of RNA sample was resuspended in tRNA resuspension buffer, 1M Na Acetate buffer, and treated with either $NaIO_4$ ('oxidized sample') or NaCl ('control sample') and incubated for 20 min at room temperature in the dark. Reactions were quenched with glucose for 15 min., then supplemented with yeast tRNAPhe (Sigma-Aldrich, Cat#R4018) and precipitated with cold ethanol overnight. Next, samples were resuspended in 50 mM tris buffer (pH = 9) and incubated for 40 min at 37°C, quenched with acetate buffer and precipitated. Finally, samples were resuspended in RNase-free water and subjected to a ligation to tRNAadap, using truncated KQ mutant T4 RNA ligase 2 (New England Biolabs, Cat. #M0373) overnight. Reverse transcription was performed with SuperScript IV reverse transcriptase (Thermo Scientific, Cat#18090050) with a CSQ_RT primer. cDNA samples were subjected to qPCR with primer pairs of ValMAC and GluMTC. For delta delta-Ct calculation, data were normalized to the yeast Phe tRNA control. The charged fraction value was calculated from a relative difference between a delta-Ct value from non-oxidized (total) and oxidized (charged) samples for each primer pair.

Statistical analysis

All data were presented as mean ± SD. Statistical analysis was performed by GraphPad Prism 9.5.0. Differences between two groups were assessed using a two-tailed unpaired Student's *t* test or one-way ANOVA followed by Dunnett's comparisons test. $P < 0.05$ was set as statistical significance.

Figure S1

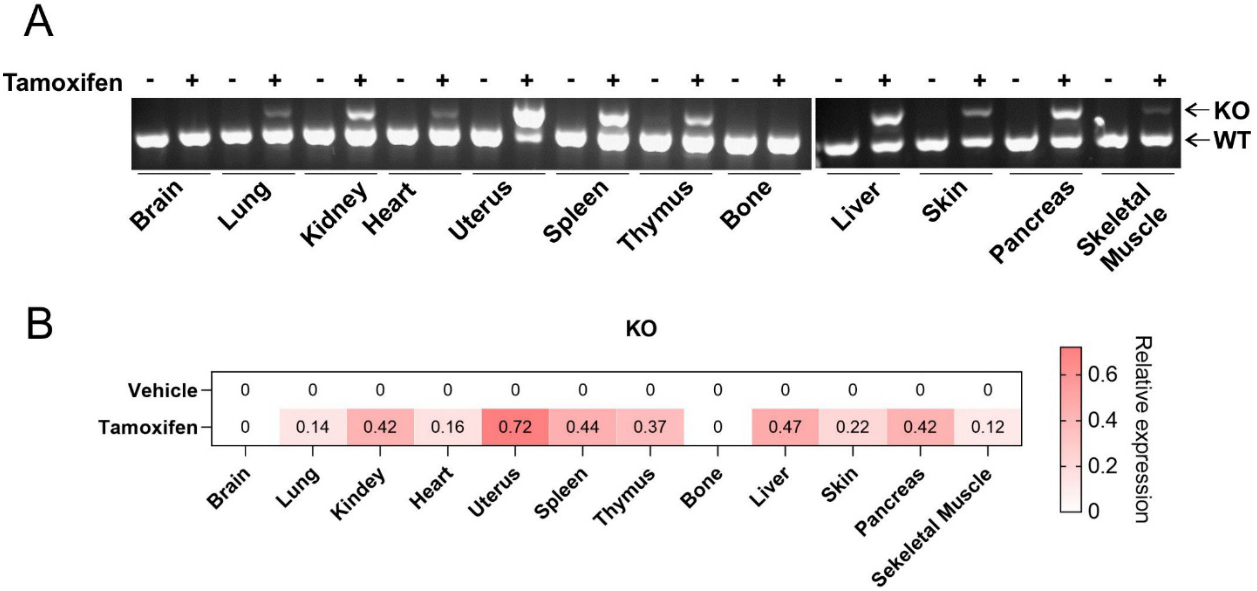

Supplementary Figure 1. Recombination efficiency across tissues following tamoxifen administration.

(A) Representative PCR analysis of genomic DNA from the indicated tissues of a WT and a TSC2 KO mouse. (B) Quantification of recombination efficiency in the indicated tissues.

Figure S2

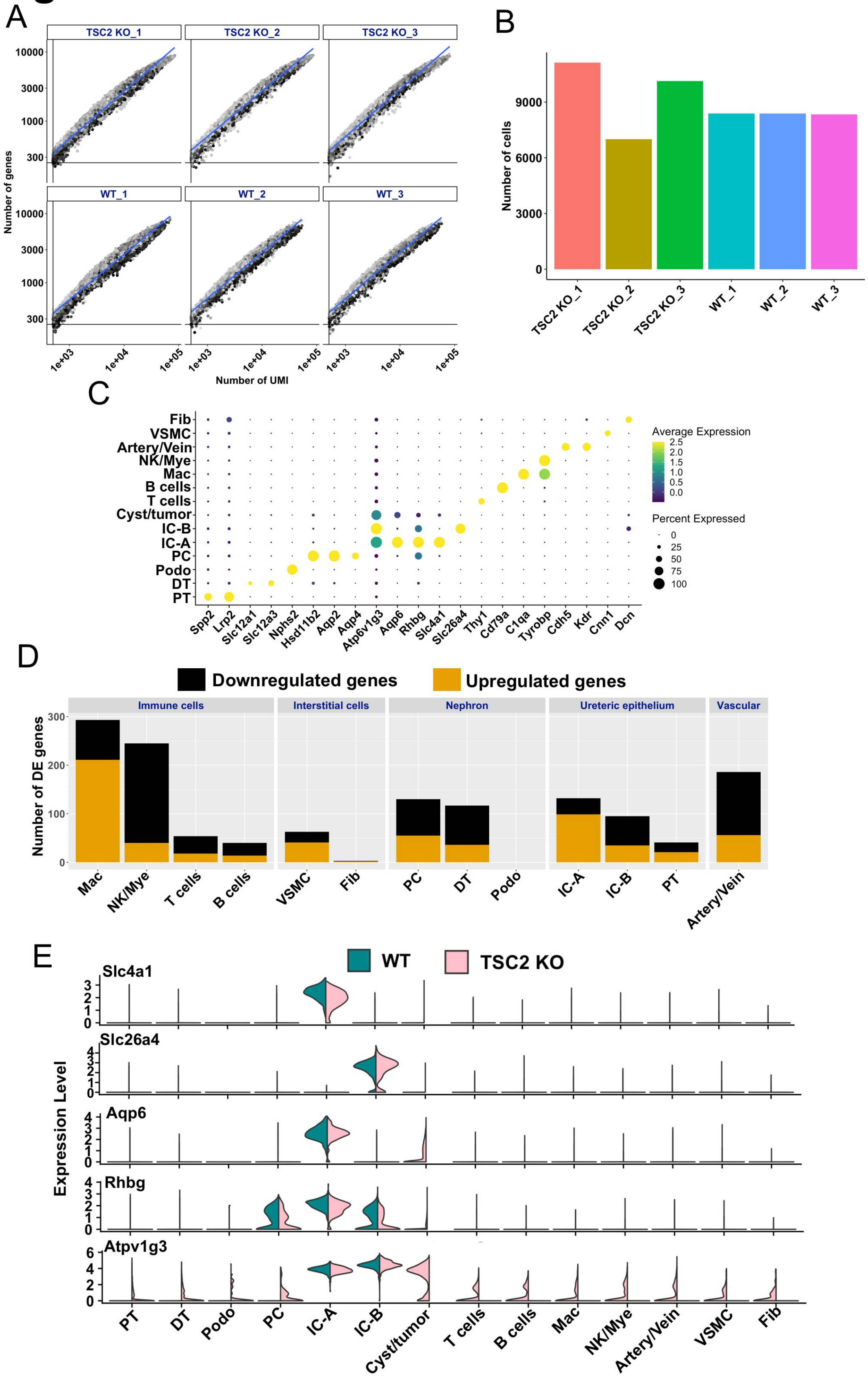

A
TSC2 KO_1
TSC2 KO_2
TSC2 KO_3
WT_1
WT_2
WT_3
Number of genes
10000
3000
1000
300
Number of UMI
1e+03
1e+04
1e+05
B
Number of cells
9000
6000
3000
0
TSC2 KO_1
TSC2 KO_2
TSC2 KO_3
WT_1
WT_2
WT_3
C
Fib
VSMC
Artery/Vein
NK/Mye
Mac
B cells
T cells
Cyst/tumor
IC-B
IC-A
PC
Podo
DT
PT
Spp2
Lrp2
Slc12a1
Slc12a3
Nphs2
Hsd11b2
Aqp2
Aqp4
Atp6v1g3
Aqp6
Rhbg
Slc4a1
Slc26a4
Thy1
Cd79a
C1qa
Tyrobp
Cdh5
Kdr
Cnn1
Dcn
Average Expression
2.5
2.0
1.5
1.0
0.5
0.0
Percent Expressed
0
25
50
75
100
D
Downregulated genes
Upregulated genes
Immune cells
Interstitial cells
Nephron
Ureteric epithelium
Vascular
Number of DE genes
300
200
100
0
Mac
NK/Mye
T cells
B cells
VSMC
Fib
PC
DT
Podo
IC-A
IC-B
PT
Artery/Vein
E
WT
TSC2 KO
Expression Level
Slc4a1
Slc26a4
Aqp6
Rhbg
Atpv1g3
PT
DT
Podo
PC
IC-A
IC-B
Cyst/tumor
T cells
B cells
Mac
NK/Mye
Artery/Vein
VSMC
Fib

Supplementary Figure 2. Quality control (QC) and differentially expressed genes of WT and TSC2 KO kidney scRNA-seq data.

(A) Scatter plots showing the correlation between the number of detected genes and UMI counts in the indicated samples. (B) Bar charts showing the number of cells after QC per sample. (C) Dot plot displaying expression levels (color intensity) and expression percent (dot size) of cluster-specific marker genes across all identified cell types. (D) Bar charts showing the number of differentially expressed genes (DEGs) in TSC2 KO versus WT samples in each cell type. (E) Violin plots showing the expression levels of Slc4a1, Slc26a4, Aqp6, Rhbg and Atpv1g3 in different cell clusters between WT and TSC2 KO mice. PC: Principal cells, Podo: Podocytes, PC: Proximal Tubule, IC-A: type A Intercalated cells, IC-B: type B Intercalated cells, NK/Mye: NK/Myeloid cells, DT: Distal Tubule, Fib: Fibroblasts, Mac: Macrophages, VSMC: vascular smooth muscle cells.

Figure S3

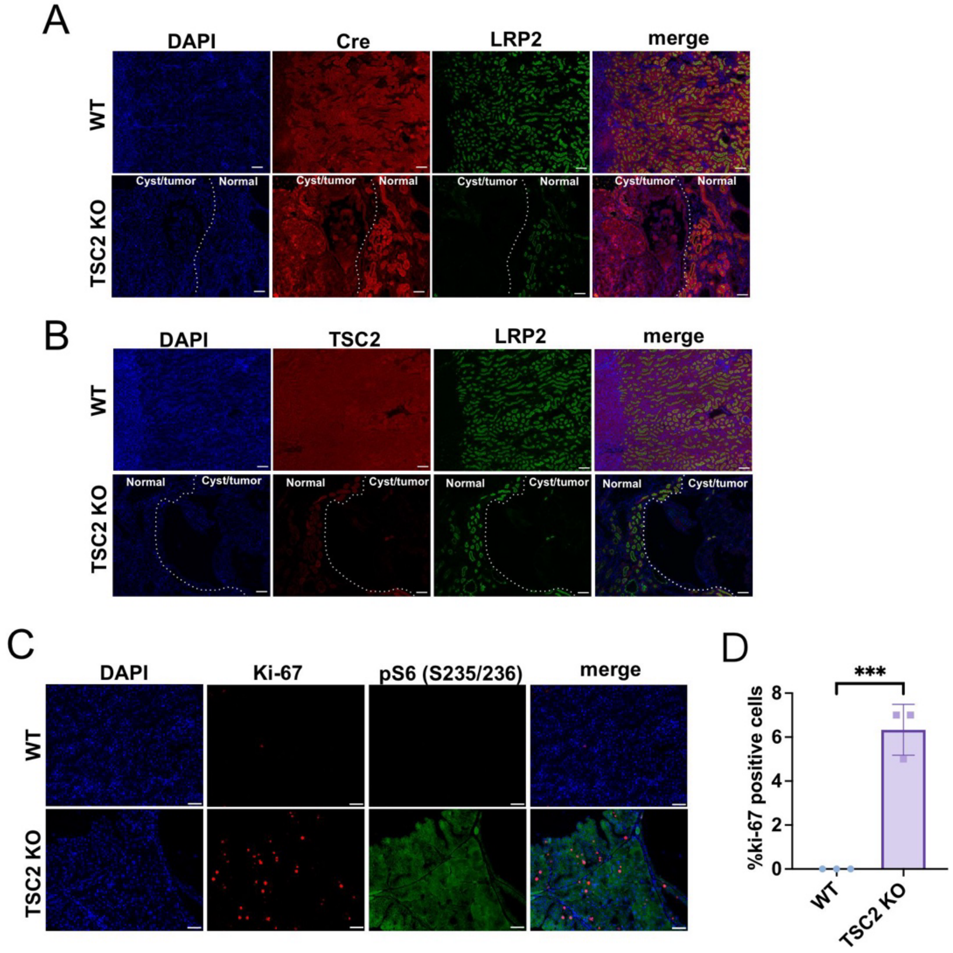

Supplementary Figure 3. Cyst/tumor cells, but not proximal tubule cells, show Cre-mediated recombination of the Tsc2 allele and increased proliferation in TSC2 KO mice.

(A) Representative images of Cre and LRP2 IF co-staining in kidney sections from mice of the indicated genotypes (n = 3). Scale bar = 100 µm. (B) Representative images of TSC2 and LRP2 IF co-staining in kidney sections from mice of the indicated genotypes (n = 3). Scale bar = 100 µm. (C) Representative images of Ki-67 and pS6(S235/236) IF co-staining in kidney sections from mice of the indicated genotypes (n = 3). Scale bar = 100 µm. (D) Quantification of the Ki-67 and pS6(S235/236) double-positive cells in WT, B7-H3 KO, TSC2 KO, and TSC2 B7-H3 dKO mice (n = 3/group). Data are presented as mean ± SD, analyzed using two-tailed unpaired Student's t-test; *** $p < 0.001$.

Figure S4

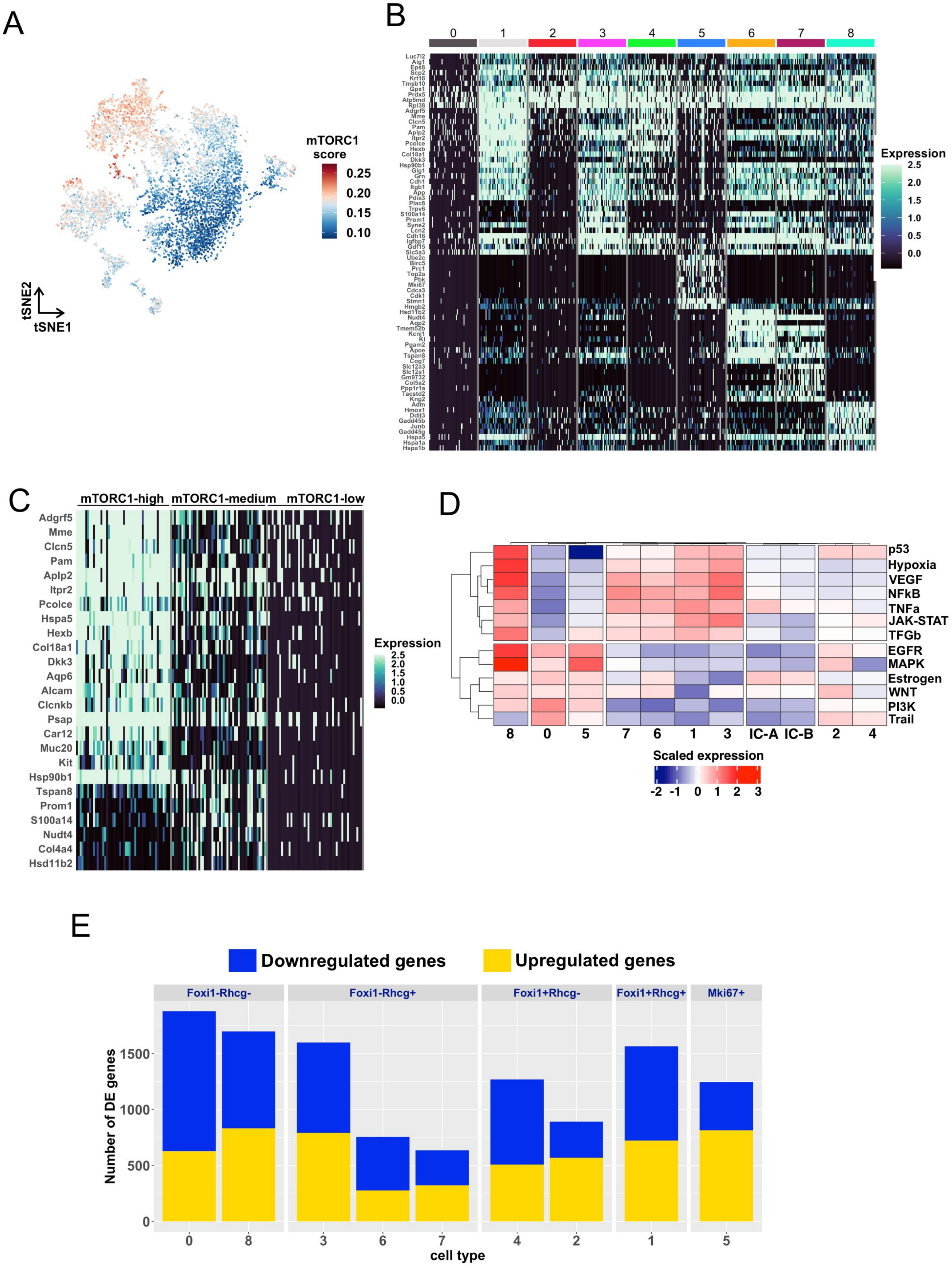
A
mTORC1 score
0.25
0.20
0.15
0.10
tSNE2
tSNE1
B
0
1
2
3
4
5
6
7
8
Expression
2.5
2.0
1.5
1.0
0.5
0.0
C
mTORC1-high
mTORC1-medium
mTORC1-low
Adgrf5
Mme
Clcn5
Pam
Aplp2
Itpr2
Pcolce
Hspa5
Hexb
Col18a1
Dkk3
Aqp6
Alcam
Clcnkb
Psap
Car12
Muc20
Kit
Hsp90b1
Tspan8
Prom1
S100a14
Nudt4
Col4a4
Hsd11b2
Expression
2.5
2.0
1.5
1.0
0.5
0.0
D
p53
Hypoxia
VEGF
NFkB
TNFa
JAK-STAT
TFGb
EGFR
MAPK
Estrogen
WNT
PI3K
Trail
8
0
5
7
6
1
3
IC-A
IC-B
2
4
Scaled expression
-2
-1
0
1
2
3
E
Downregulated genes
Upregulated genes
Foxi1-Rhcg-
Foxi1-Rhcg+
Foxi1+Rhcg-
Foxi1+Rhcg+
Mki67+
Number of DE genes
1500
1000
500
0
0
8
3
6
7
4
2
1
5
cell type

Supplementary Figure 4. TSC2 loss renal cyst/tumor cells reveal heterogeneous mTORC1-driven transcriptional programs.

(A) t-SNE plot illustrating the mTORC1 score of cyst/tumor cells calculated using UCell. (B) Heatmap displaying cluster-specific marker genes across all clusters of cyst/tumor cells. (C) Heatmap showing the expression of genes in all clusters of cyst/tumor cells associated with the mTORC1 activity score. (D) Heatmap representing the activity score of the 13 PROGENy pathways in all clusters of cyst/tumor cells, including type A (IC-A) and type B intercalated cells (IC-B). (E) Bar chart depicting the number of differentially expressed genes (DEGs) in all clusters of cyst/tumor cells.

Figure S5

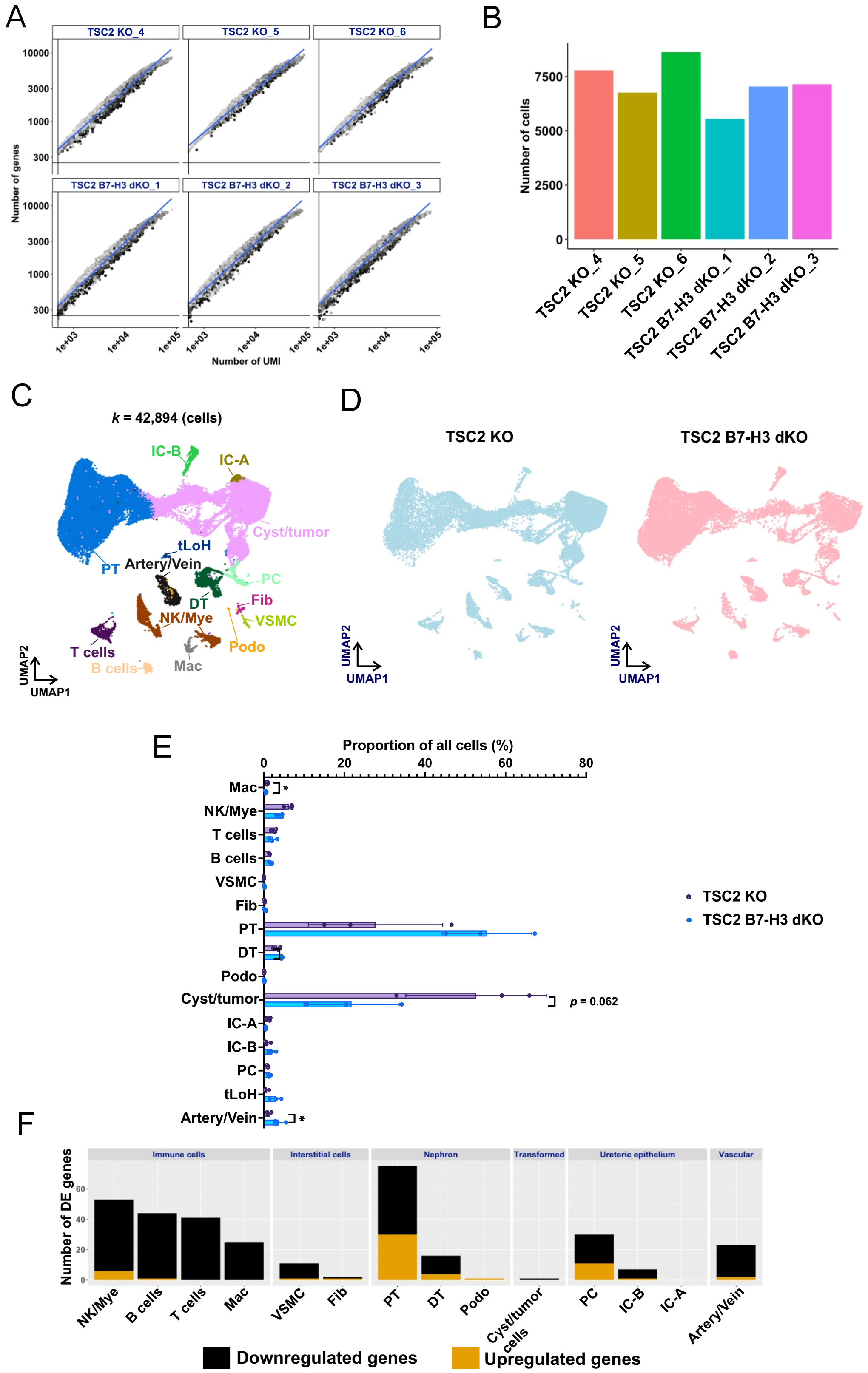

A
TSC2 KO_4
TSC2 KO_5
TSC2 KO_6
TSC2 B7-H3 dKO_1
TSC2 B7-H3 dKO_2
TSC2 B7-H3 dKO_3
Number of genes
Number of UMI
B
Number of cells
C
k = 42,894 (cells)
IC-B
IC-A
Cyst/tumor
tLoH
PT
Artery/Vein
PC
DT
Fib
NK/Mye
VSMC
Podo
T cells
B cells
Mac
UMAP2
UMAP1
D
TSC2 KO
TSC2 B7-H3 dKO
E
Proportion of all cells (%)
TSC2 KO
TSC2 B7-H3 dKO
p = 0.062
F
Immune cells
Interstitial cells
Nephron
Transformed
Ureteric epithelium
Vascular
Number of DE genes
Cyst/tumor cells
Downregulated genes
Upregulated genes

Supplementary Figure 5. Single-cell transcriptomic profiling reveals B7-H3-deficient renal cellular landscapes in TSC2 KO mice.

(A) Scatter plots showing the correlation between the number of detected genes and UMI counts in the indicated samples. (B) Bar charts showing the number of cells after QC per sample. (C) UMAP plot of a total of 42,894 cells from TSC2 KO mice and TSC2 B7-H3 dKO mice. Cells were assigned into fifteen cell types based on their marker expression. (D) UMAP atlases split by indicated genotypes. (E) Bar plot showing the comparison of the distribution of different cell clusters between TSC2 KO mice and TSC2 B7-H3 dKO mice (n = 3/group). Results are presented as mean ± SD, analyzed using two-way ANOVA with Tukey's multiple comparisons test. * $p < 0.05$. (F) Bar charts showing the number of differentially expressed genes (DEGs) in TSC2 KO versus TSC2 B7-H3 dKO samples in each cell type. PC: Principal cells, Podo: Podocytes, PC: Proximal Tubule, IC-A: type A Intercalated cells, IC-B: type B Intercalated cells, tLoH: thin Loop of Henle, NK/Mye: NK/Myeloid cells, DT: Distal Tubule, Fib: Fibroblasts, Mac: Macrophages, VSMC: vascular smooth muscle cells.

Figure S6

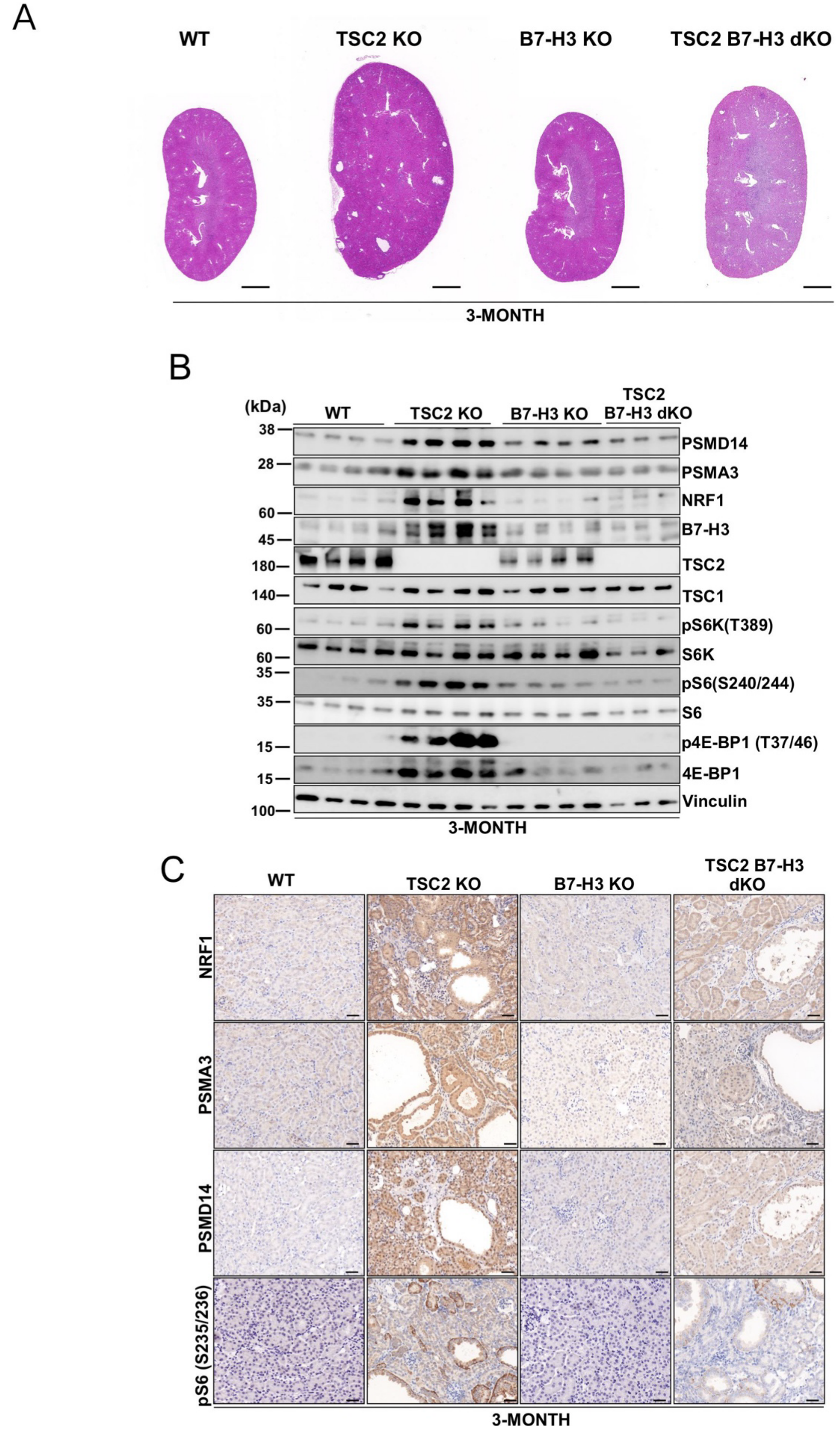
A
WT
TSC2 KO
B7-H3 KO
TSC2 B7-H3 dKO
3-MONTH
B
(kDa)
WT
TSC2 KO
B7-H3 KO
TSC2
B7-H3 dKO
38
28
60
45
180
140
60
60
35
35
15
15
100
PSMD14
PSMA3
NRF1
B7-H3
TSC2
TSC1
pS6K(T389)
S6K
pS6(S240/244)
S6
p4E-BP1 (T37/46)
4E-BP1
Vinculin
3-MONTH
C
WT
TSC2 KO
B7-H3 KO
TSC2 B7-H3
dKO
NRF1
PSMA3
PSMD14
pS6 (S235/236)
3-MONTH

Supplementary Figure 6. B7-H3 knockout reduces mTORC1 activation in 3-month-old mice.

(A) Representative H&E images of whole kidney sections from 3-month-old mice of the indicated genotypes. Scale bar = 1 mm. (B) Immunoblot analysis of whole-kidney tissue lysates from WT (n = 4), TSC2 KO (n = 4), B7-H3 KO (n = 4), and TSC2 B7-H3 dKO mice (n = 3). (C) Representative images of NRF1, PSMD14 and PSMA3 IHC staining in kidney sections from mice of the indicated genotypes (n = 3). Scale bar = 40 μm.

Figure S7

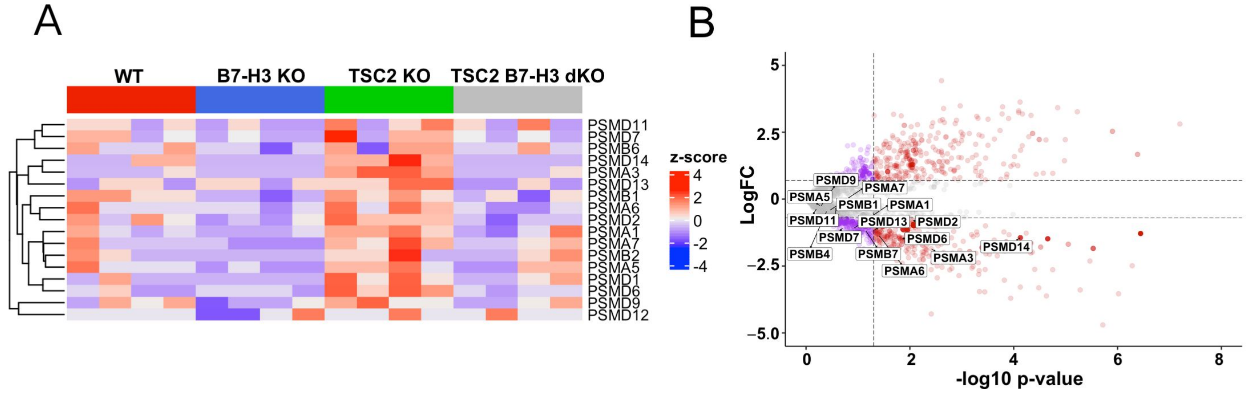

Supplementary Figure 7. Transcriptomic and proteomic profiling reveals differential expression of proteasomal protein subunits.

(A) Heatmap showing RNA expression of proteasomal protein subunits in kidney tissues from WT, TSC2 KO, B7-H3 KO and TSC2 B7-H3 dKO mice. (B) Volcano plot showing differentially expressed proteins in the kidneys of TSC2 B7-H3 dKO mice versus TSC2 KO mice. Each dot represents a protein. Proteins with absolute log2 fold-change of 1.5 and adjusted p-value $p \leq 0.05$ are represented in red.

Table S1. Primer sequences for genotyping.

R26-Cre-ER^{T2}	
Primer 1	AAA GTC GCT CTG AGT TGT TAT
Primer 2	GGA GCG GGA GAA ATG GAT ATG
Primer 3	CCT GAT CCT GGC AAT TTC G
**TSC2flox**	
Primer 1	GCA GCA GGT CTG CAG TGA AT
Primer 2	CCT CCT GCA TGG AGT TGA GT
Primer 3	CAG GCA TGT CTG GAG TCT TG
**Cd276**	
Primer 1	TGCCT CTTAC TTGGA CCACA AAG
Primer 2	CTCTG AGCAA GCCTG TGTCT
Primer 3	GGGCGCCCGGTTCTT
Primer 4	CCTCGTCCTGCAGTTCATTCA

Table S2. List of probes used for qRT-PCR.

Probe	Cat#	Company
β-actin	Mm00607939_s1	ThermoFisher Scientific
Cd276	Mm00506020_m1	ThermoFisher Scientific
Nfe2l1	Mm01299481_m1	ThermoFisher Scientific
Psmd14	Mm00451955_m1	ThermoFisher Scientific

Supplementary References

1 Adam M, Potter AS, Potter SS. Psychrophilic proteases dramatically reduce single-cell RNA-seq artifacts: a molecular atlas of kidney development. *Development* 2017; **144**: 3625–3632.

2 Fu R, Gillen AE, Sheridan RM, Tian C, Daya M, Hao Y *et al.* clustifyr: an R package for automated single-cell RNA sequencing cluster classification. *F1000Res* 2020; **9**: 223.

3 Ransick A, Lindström NO, Liu J, Zhu Q, Guo J-J, Alvarado GF *et al.* Single-Cell Profiling Reveals Sex, Lineage, and Regional Diversity in the Mouse Kidney. *Dev Cell* 2019; **51**: 399-413.e7.

4 Love MI, Huber W, Anders S. Moderated estimation of fold change and dispersion for RNA-seq data with DESeq2. *Genome Biol* 2014; **15**: 550.

5 Xing J, Du FY, Liu T, Zhu FP. Autoinduction of phase I and phase II metabolism of artemisinin in rats. *Xenobiotica* 2012; **42**: 929–938.

6 Wu T, Hu E, Xu S, Chen M, Guo P, Dai Z *et al.* clusterProfiler 4.0: A universal enrichment tool for interpreting omics data. *Innovation (Camb)* 2021; **2**: 100141.

7 Andreatta M, Carmona SJ. UCell: Robust and scalable single-cell gene signature scoring. *Comput Struct Biotechnol J* 2021; **19**: 3796–3798.

8 Lindgren D, Eriksson P, Krawczyk K, Nilsson H, Hansson J, Veerla S *et al.* Cell-Type-Specific Gene Programs of the Normal Human Nephron Define Kidney Cancer Subtypes. *Cell Rep* 2017; **20**: 1476–1489.

9 Garcia-Alonso L, Holland CH, Ibrahim MM, Turei D, Saez-Rodriguez J. Benchmark and integration of resources for the estimation of human transcription factor activities. *Genome Res* 2019; **29**: 1363–1375.

10 Schubert M, Klinger B, Klünemann M, Sieber A, Uhlitz F, Sauer S *et al.* Perturbation-response genes reveal signaling footprints in cancer gene expression. *Nat Commun* 2018; **9**: 20.

11 Friedman J, Hastie T, Tibshirani R. Regularization Paths for Generalized Linear Models via Coordinate Descent. *J Stat Softw* 2010; **33**: 1–22.

12 Street K, Risso D, Fletcher RB, Das D, Ngai J, Yosef N *et al.* Slingshot: cell lineage and pseudotime inference for single-cell transcriptomics. *BMC Genomics* 2018; **19**: 477.

13 Cao J, Spielmann M, Qiu X, Huang X, Ibrahim DM, Hill AJ *et al.* The single-cell transcriptional landscape of mammalian organogenesis. *Nature* 2019; **566**: 496–502.

14 Du H, Yang YC, Liu H-J, Yuan M, Asara JM, Wong K-K *et al.* Bi-steric mTORC1 inhibitors induce apoptotic cell death in tumor models with hyperactivated mTORC1. *J Clin Invest*; **133**: e167861.

15 Liu H-J, Du H, Khabibullin D, Zarei M, Wei K, Freeman GJ *et al.* mTORC1 upregulates B7-H3/CD276 to inhibit antitumor T cells and drive tumor immune evasion. *Nat Commun* 2023; **14**: 1214.
